# Infant mental health services for birth and foster families of maltreated pre-school children in foster care (BeST^?^): a cluster-randomized phase 3 clinical effectiveness trial

**DOI:** 10.1038/s41591-025-03534-9

**Published:** 2025-05-01

**Authors:** Karen Crawford, Robin Young, Philip Wilson, Manuela Deidda, Matt Forde, Susanne Millar, Alex McConnachie, Kathleen Boyd, Emma McIntosh, Dennis Ougrin, Marion Henderson, Christopher Gillberg, Gary Kainth, Fiona Turner, Edmund J. S. Sonuga-Barke, Bridie Fitzpatrick, Helen Minnis

**Affiliations:** 1 Centre for Developmental Adversity and Resilience (CeDAR), University of Glasgow School of Health and Wellbeing, Glasgow, UK; 2 Robertson Centre for Biostatistics (Glasgow Clinical Trials Unit), University of Glasgow School of Health and Wellbeing, Glasgow, UK; 3 Institute of Applied Health Sciences, University of Aberdeen and Department of General Practice, Institute of Public Health Science, University of Copenhagen, Glasgow, UK; 4 Health Economics and Health Technology Assessment, University of Glasgow School of Health and Wellbeing, Glasgow, UK; 5 The NSPCC, Glasgow, UK; 6 Glasgow Health and Social Care Partnership, Glasgow, UK; 7 Youth Resilience Unit, WHO Collaborating Centre for Mental Health Services Development, Centre for Psychiatry and Mental Health, Wolfson Institute of Population Health, Queen Mary University, London and East London NHS Foundation Trust, London, UK; 8 MRC/CSO Social and Public Health Sciences Unit, School of Health and Wellbeing, University of Glasgow, Glasgow, UK; 9 Department of Social Work and Social Policy, University of Strathclyde, Glasgow, UK; 10 The Gillberg Neuropsychiatry Centre and Institute of Neuroscience and Physiology University of Gothenburg and CeDAR, University of Glasgow School of Health and Wellbeing, Glasgow, UK; 11 Institute of Psychiatry, Psychology & Neuroscience, Kingʼs College London, London, UK

**Keywords:** Health care, Health services

## Abstract

Children entering foster care are at high risk of poor mental health. In this single-blind, cluster-randomized phase 3 trial, 382 families with 488 0–5-year-old children, entering foster care, were randomized to the New Orleans Intervention Model (NIM) or social work services as usual (SAU). NIM offers infant mental health assessment (~3 months) and treatment (6–9 months) to children and to their birth and foster families, aiming to improve child mental health and recommend return home or adoption. The principal outcome was child mental health, as measured by the Strengths and Difficulties Questionnaire Total Difficulties (SDQ-TD) scale at 2.5 years after study entry. In total, 286 families (149 NIM and 137 SAU, 367 children) were followed-up (79.4%). Intention-to-treat analysis found no intervention effect of NIM: mean (s.d.) SDQ-TD NIM, 11.5 (7.6); SAU, 11.1 (7.2); adjusted mean difference (NIM − SAU), 1.4; 95% confidence interval (−0.63, 3.53); *P* = 0.17. No within-trial effects for primary or secondary outcomes were observed. Despite its components being delivered to a high standard, the UK legal context surrounding NIM led to it being impossible to deliver to all eligible families, and less than 70% of families received the intervention to which they were randomized. Future research will be required to evaluate NIM in more favorable social and legal contexts. ClinicalTrials.gov registration: NCT02653716.

## Abstract (web summary)

In a trial examining the effectiveness of an infant mental health service targeting birth and foster families of children aged 0–5 years, families were randomized to receive care from either a multidisciplinary infant mental health team according to the New Orleans Intervention Model or social work services as usual. No difference in the mental health of children was found between the two arms.

## Main

Infants and pre-school children require committed family care in order to thrive^1,2^. This can be hard to achieve in families experiencing multiple challenges such as deep poverty, addictions or mental illness who are frequently marginalized and unable to access the support services they need, leading to a vicious cycle of worsening family stress^3^. If family stress becomes extreme, child protection services may need to become involved, or the child may have to come into care, while the family’s underlying difficulties often remain unaddressed^3^. Although it is well known that intervening early in a child’s life is likely to be more effective than intervening later^4^, trials of interventions with struggling families to support the development of their children and prevent child maltreatment have had limited success^5,6^.

If an infant or pre-school child is removed from the family into foster care because they are at risk of, or have experienced, child maltreatment (that is, abuse and neglect), personal, family and societal costs can be enormous: the average UK lifetime cost for every child maltreated is approximately £89,390 (ref. ^7^). Children in foster care are at high risk of neurodivergence and mental health problems, which are often longstanding^8,9^. This is perhaps unsurprising because trials of early intervention to improve the mental health and development of children in foster care found small effect sizes. Studies have almost universally focused purely on the foster family, with few aiming to also offer therapeutic intervention to birth families^10^.

To successfully support the development of young children in foster care, a multimodal approach is likely to be needed: in addition to support for the foster family to help them develop positive relationships with a child who might have mental health or relationship problems, a range of support services will be required to ascertain whether the birth family can safely have the child home. If safe return home cannot be achieved within a timescale supportive to the child’s development, a permanent alternative (that is, adoption or permanent foster care) must be found. To make these crucial decisions about a young child’s future, an authoritative family judicial system, informed by social care and infant mental health experts, will be required^11^.

We found only one intervention aiming to improve the mental health of infants and young children placed in foster care through such a multimodal approach. We call this approach the New Orleans Intervention Model (NIM) after the city in which it originated. NIM aims to improve the mental health of the infant or pre-school child by offering relationship-focused interventions targeting both the child-foster family and the child-birth family relationships. Informed by the birth family’s progress with treatment and other supports, the NIM makes a recommendation to the legal system about whether the child should return home or be adopted. NIM is delivered by a multidisciplinary infant mental health team comprising psychologists, a psychiatrist, therapists and social workers who assess the mental health and relationship quality of children younger than 5 years of age upon reception into foster care.

**Fig. 1:**
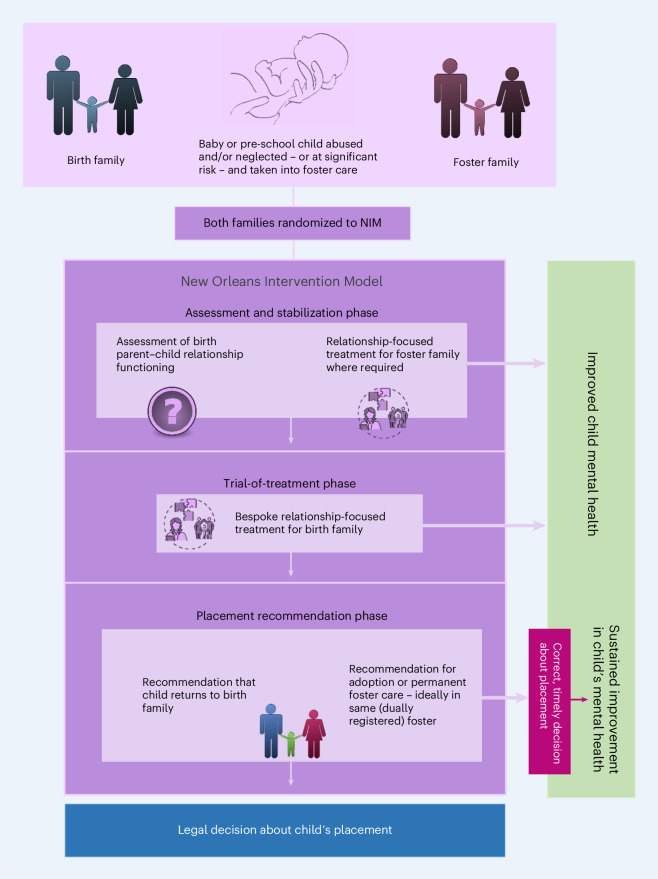
NIM is a complex intervention. Complex interventions can have several components and several actors and can target a range of behaviors^12^. NIM involves an infant or pre-school child or children, with an experience of or risk of maltreatment, with both their birth and foster family. NIM aims to improve both the child’s mental health and the decisions made about the child’s permanent placement. A timely decision about permanent return home or adoption should facilitate sustained improvements in the child’s mental health.

NIM is a complex intervention (Fig. 1), defined as such because it has several components and several actors (including birth and foster families, clinicians, social workers, other practitioners and stakeholders) and targets a range of behaviors^12^. The NIM ‘assessment phase’, involving each actual and potential caregiver, is manualized and uses structured interviews, self-report measures and observations. As assessment proceeds, where necessary, therapeutic intervention is offered, aiming to improve child–foster family relationships and child mental health. Next, in the ‘trial-of-treatment phase’, a bespoke trial of treatment is offered to the birth family (while the child remains in foster care), drawing on a small range of relationship-focused therapeutic techniques. Parents are also referred as required to other agencies for help with substance misuse, mental health issues or intra-familial violence. Lastly, in the ‘recommendation phase’, a recommendation is made to the legal system about the child’s permanent placement. Where significant positive change has been achieved, the NIM recommendation is that children are rehabilitated back to the birth family. If not, the recommendation is adoption, permanent foster care or kinship (that is, extended family) care.

Preliminary evaluation of NIM suggests success in both supporting the child’s mental health^13^ and in reducing the family’s likelihood of having to have a future child removed into care^14^. In New Orleans, several aspects of the system surrounding NIM were regarded as crucial: (1) dual registration of the foster family as a potential adoptive family so that, if the child is unable to return home, a further attachment disruption is avoided; (2) timescales appropriate to young children’s development (usually up to 15 months) within which decisions about the child’s permanent placement must be made; and (3) the entire process mandated and overseen by an authoritative family court judge.

In 2009–2010, we modified NIM for use in both Scotland and England, maintaining its core framework and attempting, as far as possible, to ensure that the impacts of any systemic differences between countries were minimized^15^. In the Best Services Trial (BeST^?^), NIM was delivered by the Glasgow Infant and Family Team (GIFT) and the London Infant Family Team (LIFT). Team training and structure were similar to New Orleans. We developed a theory of change for NIM (Fig. 1) and followed the Medical Research Council complex interventions framework^12^ to evaluate it, including conducting an internal pilot study^16^.

Embedding NIM into the UK context required consideration of similarities and differences in service delivery between the USA and the UK^15^. Services as usual (SAU) for young children coming into foster care in the UK usually follow one of three models: (1) in Glasgow, Renfrewshire and some parts of London, young children coming into foster care are allocated to a specialist team of social workers offering relationship-based assessment of parental capacity; in other London boroughs, SAU offer either (2) an assessment by the child’s allocated social worker with additional services (for example, mental health) if required, or (3) a specialist assessment carried out by an independent social worker/psychologist/psychiatrist. Regardless of the UK location, social work reports on the parental capacity assessment are submitted to the legal system for a decision to be made about whether the child can return home. In the UK as a whole, preventative childcare social work offers much more input to families in need than in the USA^15^.

There are also judicial differences between the USA and the UK and between England and Scotland. In Scotland, there are no court-mandated timescales for childcare decisions. In England, SAU are bound by a strict court-mandated 26-week timescale, whereas, if NIM treatment is deemed to be required, the court can mandate that timescales are extended up to a maximum of 40 weeks. There is no previous randomized controlled trial (RCT) evidence for NIM. This has led us to ask the following research question:

Is NIM effective in improving the mental health, relationship quality and placement stability of infants and pre-school children placed in foster care?

**Fig. 2:**
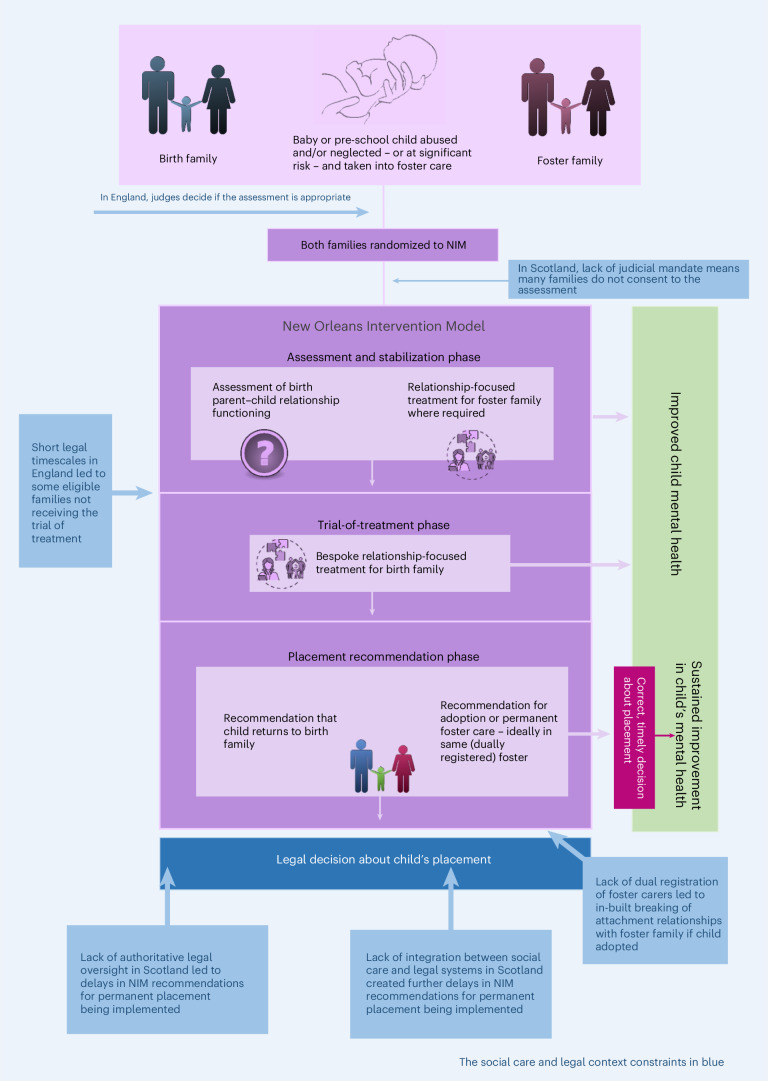
NIM in its UK social care and legal context. Medical Research Council complex interventions guidance states that implementation of a complex intervention can ‘vary across different contexts yet maintain the integrity of the core intervention components’ and that ‘Complexity might also arise through interactions between the intervention and its context’^12^.

Before conducting our main outcomes analysis, we analyzed and submitted our qualitative process evaluation of the UK trial contexts on the basis of more than 200 interviews with more than 400 participants (foster carers, birth parents, social workers, the judiciary and NIM practitioners). In brief, we found that, whereas individual components of the NIM can be delivered with fidelity in the UK, contextual constraints prevent it from being delivered as designed for all families who might benefit from it (Fig. 2).

We concluded that, for NIM to be clinically effective in the UK, major systemic changes to the systems supporting young children in foster care are required, including:

- Authoritative legal oversight with strict adherence to court-mandated processes and timescales
  ∘ No authoritative legal oversight was available in the Scottish site.
- Sufficient time allowed within legal timescales to enable virtually all families to be offered a trial of treatment
  ∘ There was insufficient time, in the English site, for all eligible families to receive a trial of treatment if randomized to NIM.
- Dual registration of foster carers as potential adopters so that if return home is not possible, the child does not need to experience a further disruption of attachment relationships
  ∘ Dual registration could not be achieved in either BeST^?^ site.
- NIM needs to be fully embedded within an integrated social care and family justice system.
  ∘ NIM was fully embedded within an integrated system in the English site but not in the Scottish site.

## Results

**Fig. 3:**
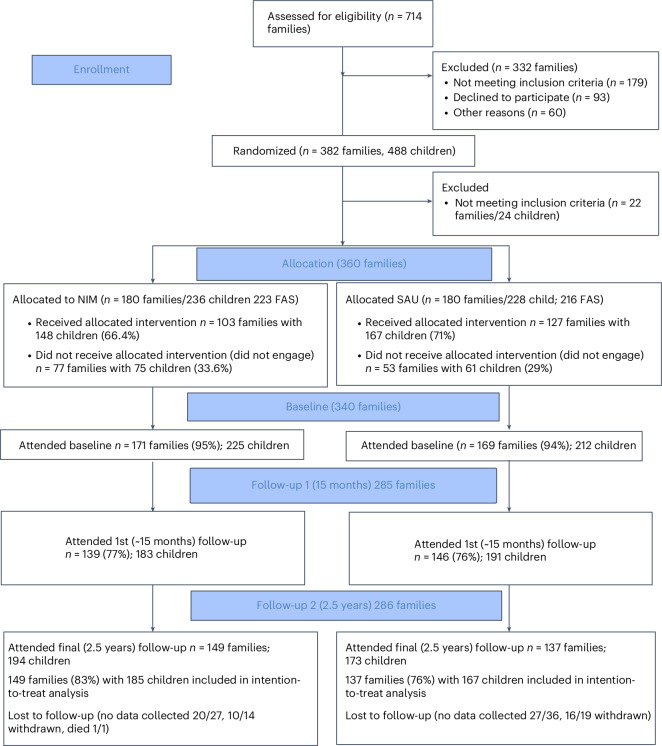
Trial profile. Study flow diagram was based on CONSORT 2010 guidance: https://www.equator-network.org/reporting-guidelines/consort/. FAS, full analysis set.

Between January 2012 and July 2021, 382 families with 488 children consented to participation in the study and were randomized 1:1 to either the NIM or SAU (see CONSORT trial profile (Fig. 3)). A total of 22 families (24 children) were excluded as ineligible shortly after randomization because the parenting capacity assessment was no longer required, usually because a Judge (England) or a Sheriff (Scotland) adjudicated that maltreatment had not occurred. In total, 360 families (464 children; 48% female) were randomized: 180 to NIM and 180 to SAU. Twenty-five children were not included in the primary analysis because they entered the study after their family was initially randomized: 360 families (439 children) comprised the full analysis set at randomization. Follow-up continued until December 2023 when the trial was fully powered: 285 families (79.2%) with 374 children were followed-up 15 months after entry to the study, and 286 families (79.4%) with 367 children were followed-up 2.5 years after entry to the study. After the 25 late-entry children were removed from the primary analysis, the full analysis set at 2.5 years was 286 families with 352 children. The intracluster correlation coefficient for the primary outcome (Strengths and Difficulties Questionnaire, SDQ) was 0.38.

Of the 223 children from 180 families randomized to NIM, 148 (66.4%) were from families deemed to be compliant with the intervention: 106 (47.5%) were fully compliant (that is, had both assessment and treatment or were not deemed to require treatment), and 42 (18.8%) were partially compliant (that is, engaged in NIM assessment only). Of the 216 children whose families were randomized to SAU, 156 (72.8%) started an assessment (see Supplementary Table 2 for details).

For the Glasgow cohort (*n* = 338 children, broadly representative of the general population of looked-after children in Glasgow^17^), data were available on pre-care abuse and neglect: 65% had experienced emotional neglect, 57% domestic violence, 48% emotional abuse, 19% physical abuse and 2% sexual abuse, and 56% had a parent with a severe mental illness, 36% had a parent who had been incarcerated and 63% had a parent who had problems with substance abuse. For a representative subsample of Glasgow children (the first 198), we scrutinized social care data for placement numbers and types (Supplementary Table 3): the median number of care placements was 3 (range, 1–9). Just over half (55.6%) of the sample eventually achieved legal permanence, although this sometimes took much longer than 2.5 years. Permanent return to birth family was the most common form of permanence (22.2%), followed closely by adoption (21.2%). The mean (s.d.) commitment (This is My Baby (TIMB) ratings) of the foster carers to the children in their care, at baseline, was similar to that in similar US samples: 3.74 (0.94) compared to 3.8 (1.2)^18^. Of the children aged 2 years and older (Glasgow and London) whose foster carer completed a Development and Wellbeing Assessment (DAWBA) at baseline (*n* = 139), 40% had at least one psychiatric or neurodevelopmental condition, including 10.1% with an anxiety disorder, 3.6% with post-traumatic stress disorder, 2.2% with a depressive disorder, 8.6% with attention-deficit/hyperactivity disorder (ADHD), 8.6% with autism, 11.5% with oppositional defiant or conduct disorder, 7.2% with reactive attachment disorder (RAD) and 15.1% with disinhibited social engagement disorder (Table 1 and Supplementary Table 4).

**Table 1:** Baseline characteristics of families (*n* = 360 families and *n* = 439 children)

	Intervention group, NIM (*n* = 223 children)	Control group, SAU (*n* = 216 children)
*n* (%) or mean (s.d.) (numbers vary slightly due to missing data)		
Maternal age (mean) Paternal age (mean)	29.1 33.3	28.3 32.7
Index for Multiple Deprivation (%)		
Quintile 1	161 (80.9)	145 (77.5)
Quintile 2	24 (12.1)	26 (13.9)
Quintile 3	10 (5.0)	8 (4.3)
Quintile 4	2 (1.0)	3 (1.6)
Quintile 5	2 (1.0)	5 (2.7)
Care episodes before first in RCT (%)		
0	151 (86.3)	139 (83.7)
1	22 (12.6)	25 (15.1)
2	2 (1.1)	2 (1.2)
Care order type (%)		
Voluntary	88 (40.6)	92 (43.4)
Compulsory	129 (59.4)	120 (56.6)
Mean age (s.d.; range) at baseline (years)	2.00 (1.64; 0.01–5.38)	1.98 (1.65; 0.01–5.40)
Sex (% female)	96 (43.4%)	108 (50.7)
Ethnicity (%)		
White	172 (78.5)	173 (82.8)
Mixed	21 (9.6)	18 (8.6)
Asian or Asian British	10 (4.6)	6 (2.9)
Black or Black British	15 (6.8)	12 (5.7)
Other ethnic group	1 (0.5)	0 (0)
Reasons for coming into care (%)		
Physical abuse	34 (15.4)	28 (13.1)
Emotional abuse	39 (17.6)	16 (7.5)
Neglect	114 (51.6)	114 (53.5)
Experience of domestic abuse	60 (27.1)	64 (30.0)
Parent substance/alcohol abuse	110 (49.8)	102 (47.9)
Parent mental illness	73 (33.0)	49 (23.0)
Homeless or inadequate home	35 (15.8)	28 (13.1)
(70.5% of children had more than one reason for coming into care)		
Mean (s.d.) SDQ-TD: reference 7.5 (5)	11.8 (8.0) *n* = 94	14.0 (8.1) *n* = 84
Mean PIRGAS score	81.7 (12.2) *n* = 148	79.2 (14.9) *n* = 139
Mean PedsQL score: reference 87.6 (12.3)	87.3 (11.9) *n* = 119	87.4 (12.2) *n* = 187

### Primary outcome

When comparing children randomized to NIM with children randomized to SAU on the primary outcome (child mental health as measured by the Strengths and Difficulties Questionnaire Total Difficulties (SDQ-TD)), no statistically significant intervention effect of NIM compared to SAU (Table 2) was observed.

**Table 2:** Primary and secondary outcomes 2.5 years after randomization

	Intervention group, NIM (*n* = 185 children)	Control group, SAU (*n* = 167 children)	Mean difference (or HR)	95% CI	*P* value
**Primary outcome**					
SDQ-TD score^a^	11.5 (7.6)	11.1 (7.2)	1.44	(−0.63, 3.53)	0.17
**Secondary outcomes**					
Time (years) to permanent legal status (HR)^b^	1.38 (0.67)	1.32 (0.73)	0.98	(0.68, 1.43)	0.93
PIRGAS score^a^	83.66 (12.73)	84.83 (9.85)	−2.82	(−8.53, 2.84)	0.33
PedsQL score^a^	87.2 (12.3)	87.3 (12.4)	−0.05	(−0.27, 0.16)	0.63

^a^Effect estimates, 95% CIs and *P* values were derived from generalized linear mixed effects regression models for the interaction of treatment at each visit, adjusting for age, family status at randomization (solo child versus multiple), sex, site (Glasgow versus London), age-appropriate questionnaire version (PedsQL only) and random effects for family structure. Comparisons are for the intervention (NIM) group compared to the control (SAU) group at the final follow-up visit.

^b^HRs, 95% CIs and *P* values come from a Cox proportional hazard model including adjustments for age, family status at randomization (solo child versus multiple), sex and site (Glasgow versus London). Comparison is given for ‘survival’ (that is, continued status in temporary care) of the SAU group compared to the NIM group.

All *P* values are two-sided. No adjustments were made for multiple comparisons.

### Secondary outcomes

When comparing children randomized to NIM with children randomized to SAU on the secondary outcomes (Pediatric Quality of Life Inventory (PedsQL), Parent–Infant Relationship Global Assessment (PIRGAS) and time to permanent placement), no statistically significant intervention effect of NIM compared to SAU (Table 2) was observed.

### Safety

There were 52 serious adverse events (SAEs; 21 in the NIM arm and 31 in the SAU arm) in 41 participants. Twenty-four deaths (10 in the NIM arm and 14 in the SAU arm) included 23 parental deaths and one sudden infant death. Similar numbers of SAEs were identified through research assessments and administrative data, and 56% of SAEs were identified through both means. No SAEs were judged to be intervention related.

### Pre-specified exploratory outcomes

No difference was observed between NIM and SAU in the proportions of children for whom the recommendation was permanent placement away from home (28% NIM and 29% SAU; Supplementary Table 3). In addition, no interaction was observed between study site and arm of trial (Supplementary Table 5). There was, however, a significant interaction (*P* = 0.0217) between sex and arm of trial such that girls randomized to NIM had significantly poorer SDQ scores compared to SAU (2.96; 95% confidence interval (CI) (0.12, 5.82)), whereas boys did not (0.12; 95% CI (−2.82, 3.09)) (Supplementary Table 6). Regardless of arm of trial, girls had significantly higher PIRGAS scores than boys (mean difference, 3.81; 95% CI (1.11, 6.53); *P* = 0.0061), but no significant interaction was observed between sex and arm of trial for PIRGAS (*P* = 0.1438) (Supplementary Table 7).

Examining the cohort as a whole, whether randomized to NIM or SAU, it was less likely that older children (hazard ratio (HR) = 0.88; 95% CI (0.78, 1.00)) and children who entered foster care along with their sibling(s) (HR = 0.56; 95% CI (0.36, 0.87)) would be settled in a permanent placement with a legal order 2.5 years later—either in an alternative placement with a permanent legal order, usually adoption, or home with the birth family without legal orders compelling social work supervision. A large and statistically significant difference was observed in the rate at which children were placed permanently with a legal order across 2.5 years in England versus Scotland: HR = 4.36; 95% CI (2.96, 6.40); *P* < 0.0001.

### Sensitivity analysis for those complying with the intervention only (CACE analysis)

When comparing the intervention effect for the 66.4% of birth families who complied with the NIM with the intention-to-treat effect estimated for all families randomized to the NIM, effect sizes were similar: intention-to-treat (0.32; 95% CI (−1.21, 1.85)); compliers only (0.43; 95% CI (−1.65, 2.51)).

### Post hoc analyses

Bang’s index (Supplementary Table 1) suggested that researcher blinding to arm of trial was successful at 2.5 years.

## Discussion

BeST^?^ set out to examine the effectiveness of an infant mental health service (NIM) that targeted birth and foster families of children aged 0–5 years and made recommendations to the legal system about children’s permanent placements. The primary outcome was the mental health of children randomized to the NIM compared to those receiving SAU. We recruited 488 0–5-year-old children, from 382 families, coming into an episode of foster care in Scotland or England. Participating families were randomized to receive a parenting capacity assessment from either a multidisciplinary infant mental health team, NIM, or social work SAU. We followed-up 79.4% of families and reported on their 367 children 2.5 years later.

Despite this trial being large and well powered (approximately twice the size of previous RCTs targeting young children in foster care), no statistically significant intervention effect was observed of NIM on child mental health as measured by the SDQ-TD nor as measured by our secondary outcomes of quality of life (PedsQL), child–carer relationship quality (PIRGAS) or time taken to permanent legal status (TTPLS).

This contrasts starkly with positive findings documented in some previous trials of interventions aiming to improve the mental health and development of young children in care^19^. For example, the Bucharest Early Intervention Project (BEIP), despite being a much smaller RCT, evidenced major developmental gains for children randomized to the intervention group^20^. This is perhaps unsurprising because children in the BEIP were randomized into a radically different care environment (foster care versus poor-quality institutional care), whereas, in BeST^?^, most children remained in temporary foster care during the 2.5 years after randomization to NIM or SAU.

We developed strong multi-agency partnerships to support BeST^?^, and each component of the NIM was delivered with fidelity and to a high standard. Despite this, NIM could be delivered to only 66.4% of eligible families. The ‘…fairly radical changes in approaches and attitudes towards permanency planning for maltreated children’ that we had identified in 2010 as being required for NIM to be successfully delivered in the UK^15^ were not achieved, especially in the Scottish site.

Our target population of infants and pre-school children had much higher levels of mental health needs than their peers in the general population: the 40% prevalence of psychiatric disorders that we found at baseline is approximately five times greater than that in the general population of infants and pre-schoolers, in which prevalence estimates range from 7% to 9%^21,22^. During the study, there were 24 parental deaths and one child death—a mortality rate similar to other high-risk populations but much higher than the general population^23^. It is extremely unfortunate that the complexities of our UK social care and judicial systems do not support the delivery of mental health and social care services to the families who most need them. It will be important, in future research, to evaluate NIM within social care/judicial systems similar to that in New Orleans, with authoritative judicial oversight of the process and timescales long enough for the NIM treatment phase to be delivered to all families who require it.

Despite our overall null findings, we found a significant intervention effect for girls, with those randomized to NIM having higher SDQ-TD scores at 2.5 years than those randomized to SAU. We would like to mention two potential plausible mechanisms. First, girls may have benefitted from the (usually) faster decision-making about placement achieved through SAU (parenting capacity assessment completed within approximately 3 months compared to approximately 1 year for NIM). However, any negative mental health impact of longer NIM timescales on girls might be mitigated in other jurisdictions by dual registration of foster carers as adopters, because the child’s permanent placement (through return home or adoption) would always be with a family that is already fully committed to their long-term development^1^. The second plausible mechanism for the high SDQ-TD scores in girls randomized to NIM could have an opposite interpretation: as young children develop through toddlerhood and into the pre-school period, attachment behaviors are expressed at times of distress by noisy cries and physical signaling—for example, reaching out to or running after a caregiver—and, when young children’s needs are frustrated, temper tantrums are common^24^. It is possible that, as attachments form and young children begin to feel comfortable expressing their feelings, foster carers might notice more emotional and behavioral difficulties as these become overtly expressed. We previously showed that children, in this sample, who were placed with more highly committed foster carers were more likely to have a reduction in RAD symptoms over the first 15 months but to have higher SDQ-TD scores at 2.5 years^17^. This, and our qualitative exploration with foster carers and infant mental health clinicians^1^, led us to speculate that increased SDQ-TD scores might indicate a process of recovery through which the child’s behavior gets worse before it gets better.

Regardless of trial arm, we found a reduced likelihood that older children, or those placed with their siblings, would be permanently placed (at home or in adoption or foster/kinship care) at 2.5 years. Both of these factors were found, in previous research, to be associated with placement delays and instability^25^. Over the 2.5 years after entering the study, the rate at which children were placed permanently was more than four times higher in London than in Glasgow. In England, parenting capacity assessments are mandated by the Family Court Judge, and processes leading to crucial decisions determining a child’s future family life are governed by strict timescales^11^. We concluded, in our a priori process evaluation, that such authoritative legal oversight and full embedding in an integrated social care and legal system were essential for the NIM to operate effectively within the UK context.

Despite contextual differences surrounding NIM in New Orleans versus the UK (for example, dual registration of foster carers as adopters and strict legally mandated timescales) being recognized before the study began, we were unable to influence changes to these contextual challenges during the lifetime of the study, resulting in only 66.4% of families being able to comply with NIM. Although NIM aimed to influence decisions about whether the child could go home or be adopted, this study was not designed to examine whether such decisions were correct for any specific child, their birth family or their adoptive family.

Smaller numbers of families recruited in London limited our ability to examine subgroup differences by site, and, because of heterogeneity across study sites, future trials will be required to determine whether NIM might be effective in certain contexts. Similarly, future studies will be required to examine the impact of compliance issues on outcomes of the NIM, because the complier average causal effect (CACE) analysis had similar effect sizes to the intention-to-treat findings. Challenges in obtaining video data resulted in low completeness of PIRGAS throughout the study; however, a comparison of demographic data between those with and without a PIRGAS suggests that there was little systematic bias, except a mean age approximately 5 months older in those with a PIRGAS compared to those without (Supplementary Table 8). Time to permanent placement data were censored at 2.5 yearsʼ follow-up, but we know that some children who were rehabilitated home later came back into care. However, the large numbers recruited and retained compared to other RCTs involving children in foster care, and the consent of virtually all participants for routine data future follow-up or being approached for future studies, means that the uncertainties regarding intervention effects and mechanisms can be examined later in children’s lives.

This trial has some important implications for policymakers. The stark differences that we have evidenced in rates of permanent placement in Scotland versus England, and the lower proportions of families accessing NIM treatment in England, demonstrate that the nature of the systems supporting children in foster care might have affected our ability to deliver infant mental health interventions. Previous studies, such as the BEIP, showed that radical system changes facilitating early interventions being delivered to high-risk children can markedly improve their developmental outcomes^20^. Our compliance and process findings suggest important implications for policy: if mental health services are to have the chance of being effective, we must:

1. Allow enough time within judicial timescales for treatment to be offered.
2. Reduce unnecessary delays in the system so that clinical recommendations can be enacted within timescales that support the child’s healthy development.

Globally, future RCTs must be conducted to address the stark evidence gap regarding effective mental health interventions for children in care^19^. This should include RCTs examining radical system changes, such as in the BEIP^20^, and could usher in a new era of RCTs conducted within family justice systems^4^. Follow-up of this cohort will be crucial to investigate whether intervention effects on girls generalize out to boys and to elucidate whether either of our proposed mechanisms can be supported.

Despite its components being delivered to a high standard, the legal context surrounding the NIM in England and Scotland led to the NIM being impossible to deliver to all eligible families. We observed no significant difference in children’s mental health whether randomized to NIM or SAU.

## Methods

### Study design and participants

We conducted a multi-site, pragmatic, parallel group, single-blind (researchers blinded to arm of trial), cluster-randomized controlled superiority trial with an allocation ratio of 1:1 to compare the NIM for infant mental health to social work SAU (NCT01485510) (see also the published protocol^26^). The trial was approved by West of Scotland Research Ethics Service, Committee 3 (15/WS/0280).

#### Inclusion criteria

Families were eligible for the trial if they had a child aged 0–60 months who entered care in the recruiting sites for reasons associated with maltreatment.

#### Exclusion criteria

Families were excluded from the trial if the parent(s) was unavailable to take part in an intervention (for example, because of death, unknown whereabouts or long-term imprisonment).

### Study settings and context

The trial was conducted in two large UK cities, both containing areas of material deprivation. The study settings were social services in the local authorities/boroughs feeding into the two trial sites: Greater Glasgow and Clyde (Scotland), comprising Glasgow City Council and Renfrewshire Council, and London (England), including the boroughs of Croydon, Tower Hamlets, Sutton, Bromley and Barking & Dagenham. The social and legal contexts in the two sites varied considerably: in Scotland, legal decisions regarding children’s care placements are largely made by a Children’s Panel of lay members, although decisions about the presence/absence of maltreatment, and final adoption decisions, are made judicially by a Sheriff; in England, all legal decisions about children’s care placements are made within a court setting, presided over by a Judge^11^.

### Randomization and masking

Random allocation was performed, through a web portal requiring a login and password with access rights allocated to relevant Clinical Trials Unit staff, using a mixed minimization/randomization method, stratified within the study site using an a priori schedule for each site in blocks of 10. Minimization factors were those likely to impact delivery of the NIM: study site, age of youngest child coming into care at randomization (<2/≥2 years), number of children coming into care at randomization (1/>1), birth family fluency in English and type of care (that is, foster or kinship (that is, with extended family)). Masking was assured for two secondary outcome measures, TTPLS and PIRGAS, because data were collected (TTPLS) and rated (PIRGAS) by individuals with no contact with participants or other trial procedures. Participants were aware of the intervention to which they had been allocated; data collectors, researchers and statisticians were not. Data collectors used a script, when conducting research assessments, aiming to ensure that participants did not reveal their group allocation, and, after assessment, data collectors recorded the intervention to which they thought the participating family was allocated. Trial group assignment was made available only when data collection was complete and after database lock.

### Procedures

NIM (see panel) is an infant mental health intervention delivered by a multidisciplinary health and social care team. Families were eligible if they had a child aged 0–60 months entering care in the study sites for reasons associated with maltreatment. Children in eligible families entering care subsequently were allocated (rather than randomized) to the same intervention as the family had originally received. Families were excluded if parents could not receive interventions because of, for example, death or long-term imprisonment.

We used a novel recruitment and retention method: experienced social workers (former team leaders) screened social care records for eligibility and conducted information and consent meetings with interested participants. From January 2012 to June 2015, participants were asked for consent, assessed at baseline and then randomized. Thereafter, certain changes to randomization procedures were necessary (Supplementary Table 9): to give participants more time to consider participation, between June 2015 and September 2017 randomization was conducted first, and written deferred informed consent was obtained as soon as possible after randomization and before baseline assessment^26^ (Supplementary Table 9a). This caused further delays and problems with NIM capacity, and, when the English site joined the study, the only legally justifiable option was for families to be consented and randomized before baseline assessment^11^.

### Outcomes

Study measures are detailed and referenced in Supplementary Table 10. Because NIM aims to improve the mental health of the infant or pre-school child by offering relationship-focused interventions making a recommendation to the legal system about the child’s permanent placement (Fig. 1), primary and secondary outcome measures focused on mental health/quality of life, relationship functioning and permanency. The primary outcome measure was the 20-item SDQ-TD scale at 2.5 years after study entry, assessed at each site. The SDQ is a mental health screening questionnaire for 2–16-year-old children, completed by the primary caregiver, with 25 items in five subscales: emotional symptoms, conduct problems, hyperactivity/inattention, peer relationship problems and prosocial behavior. The SDQ-TD does not include the prosocial subscale and contains 20 items. Higher SDQ-TD scores indicate poorer child behavior^27^.

Secondary outcome measures were as follows:

- Child quality of life (PedsQL)^28^
- TTPLS being achieved, authorizing the child to be placed in permanent care (return home without social work oversight or adoption)^26^
- Quality of the child’s relationship with the primary caregiver (PIRGAS). For PIRGAS, a short playtime and mealtime video was conducted in the research clinic and independently rated by specifically trained raters^29^.

Study data were collected following standard operating procedures by trained staff. For interview and video measures requiring rating, more extensive training was provided until raters reached sufficient reliability, and then inter-rater reliability was checked on 10–20% of randomly selected tests, and, if insufficient agreement, ratings were brought to an approximately three-monthly conference with an expert rater (Supplementary Table 11). Other measures included the DAWBA for psychiatric diagnosis and the TIMB interview for foster carer commitment. TTPLS and the child’s pre-care history of abuse and neglect were established by experienced social worker researchers scrutinizing each child’s social work casefile using the Adverse Childhood Experiences questionnaire and the Modified Maltreatment Classification System^30^. Data were collected at baseline (4–14 weeks after care entry), approximately 15 months after care entry and 2.5 years after randomization—face to face until March 2020 and then via telephone.

SAEs were recorded at research assessments, and, owing to concern about the level of risk in this population, a linkage to administrative data was made in November 2020.

### Statistical analyses

The sample size was calculated by the Glasgow Clinical Trials Unit. Initially, the target sample size was 462 to achieve 90% power to detect an effect size of 0.35 s.d. on SDQ-TD with a loss to follow-up at 2.5 years of 25%^26^. With no information as to the likely degree of clustering of the primary outcome within families, we aimed to recruit 462 families, even though the unit of analysis would be the individual child. Although we later decided to constrain the sample size for financial reasons^26^, follow-up rates proved better than expected, and the trial was well powered. Given the s.d. of SDQ-TD observed in the trial at 2.5 years, a 0.35 s.d. difference equates to a clinically meaningful 2.5-point difference in the 20-item SDQ-TD scale.

A statistical analysis plan was prepared before database lock (see Supplementary Information). Clustering was at the level of the family because, if the family had more than one maltreated child, any effects of the NIM were likely to have an impact on every child. The primary analysis, conducted in R version 4.1.1 (2021-08-10) for Windows (with ‘lme4’ and ‘survival’ for TTPLS)^31,32^, used a generalized linear mixed effects regression model for the primary outcome measure (SDQ-TD), with random effects terms for both family and child, to account for clustering of outcomes within families and adjusting for child age and other minimization factors. All models were adjusted for age at measure completion. A single model over all three timepoints was fitted, and interaction terms were used to estimate intervention effects at each timepoint. Models were extended to include additional interaction terms to assess whether intervention effects varied between subgroups, for the following planned subgroup analyses for which sample size was sufficient: study site and sex. The primary analysis was intention-to-treat. To examine the causal effect of the NIM intervention in the presence of non-compliance, a CACE analysis was also conducted (using Applied Econometrics with R)^33^. This was achieved by comparing outcomes for individuals in the NIM group who complied with treatment (Supplementary Table 2) to individuals receiving SAU who were randomized to the NIM and would have complied with the NIM had they been given the opportunity to do so. A sensitivity analysis examined intervention effects for index children only. Bang’s unblinding index^19^ was used (using BI)^19^ to examine unblinding success.

We used the CONSORT framework to report the findings (see the checklist in Supplementary Information).

### Reporting summary

Further information on research design is available in the Nature Portfolio Reporting Summary linked to this article.

## Online content

Any methods, additional references, Nature Portfolio reporting summaries, source data, extended data, supplementary information, acknowledgements, peer review information; details of author contributions and competing interests; and statements of data and code availability are available at 10.1038/s41591-025-03534-9.

## Supplementary information

Supplementary Materials, including Supplementary Tables 1–16, the statistical analysis plan and some additional analyses

Reporting Summary

## Data Availability

Data requests will be considered by the Trial Management Group (TMG), which includes representatives of the sponsor, the University of Glasgow, senior investigators independent of the research team and the chief investigator. The TMG will take account of the scientific rationale, ethics, logistics and resource implications. Data access requests should be initially submitted by email to the chief investigator (H.M., corresponding author). The source data, including the de-identified numerical data used for the statistical analyses sufficient to replicate primary and secondary analyses, will available within 6 months of publication. Data access will be provided through the secure analytical platform of the Robertson Centre for Biostatistics. This secure platform enables access to de-identified data for analytical purposes, without the possibility of removing the data from the server. Requests for transfer of de-identified data will be considered by the Steering Group, and, if approved, a collaboration agreement would be expected. The TMG will consider any cost implications, and cost recovery would be expected on a not-for-profit basis.
