# Supplementary material for: Infant mental health services for birth and foster families of maltreated pre-school children in foster care (BeST?): a cluster-randomized phase 3 clinical effectiveness trial
Source: Nat Med. 2025 May 1;31(5):1617–25. doi: 10.1038/s41591-025-03534-9 (PMC12092239; doi:10.1038/s41591-025-03534-9)
Supplement: Supplementary file 1 — Supplementary Materials, including Supplementary Tables 1–16, the statistical analysis plan and some additional analyses [file 41591_2025_3534_MOESM1_ESM.pdf]

nature medicine

Supplementary information

https://doi.org/10.1038/s41591-025-03534-9

Infant mental health services for birth and foster families of maltreated pre-school children in foster care (BeST?): a cluster-randomized phase 3 clinical effectiveness trial

In the format provided by the authors and unedited

Supplementary materials

Contents

Blinding....2
Compliance with NIM and SAU....3
Placement Types....4
Psychiatric Diagnoses at baseline....5
Pre-specified subgroup analyses....6
PIRGAS Sample Representativeness....9
Randomisation conditions....10
Study Measures....12
Inter-rater reliability of PIRGAS and TIMB....14
Statistical Analysis Plan....15
1. Introduction....15
1.1. Study Background....15
1.2. Study Objectives....15
1.3. Outcomes....16
1.3.1. Primary Outcome....16
1.3.2. Secondary Outcome....16
1.3.3. Other Outcomes....16
1.4. Study Design....17
1.5. Randomisation and Consent....17
1.6. Sample Size and Power....17
1.7. Study Population....18
1.8. Statistical Analysis Plan (SAP)....18
1.8.1. SAP Objectives....18
1.8.2. General Principles....18
1.8.3. Current Protocol....19
1.8.4. Deviations To Those Specified In The Protocol....19
1.8.5. Software....19
2. Analysis....20
2.1. Study Populations....20
2.2. Baseline Characteristics....20
2.3. Efficacy Outcomes....21
2.3.1. Primary Outcome....21
2.3.2. Secondary Outcomes....21

2.4. Safety Outcomes 21
2.4.1. Serious Adverse Events 21
2.4.2. Withdrawal of Consent 22
2.5. Subgroups 22
2.6. Additional Analyses 22
3. Document History 23
4. Tables 23
5. Figures 23
6. Listings 23
Index children 24
Additional analyses 24
Reporting checklist for randomised trial 31

Blinding

Supplementary Table 1 – Bangs Index

A cross tabulation of actual treatment groups (columns) is given against guessed treatment by randomised participants (rows). Bangs index is calculated for each treatment group separately. In general, if -0.2 ≤ Bang Index ≤ 0.2, blinding is considered to be successful. The vast majority of responses suggest that there was no idea of what treatment group families/children were randomised to. Where a guess of assessment group has been made there is some suggestion that it was known to be the NIM group, when this was the true randomisation, whereas guesses were less likely to be correct if randomisation was to SAU.

**Visit 1**		
	NIM	SAU
NIM	12	1
SAU	2	7
No idea	113	100
**Bangs Index**	**Estimate**	**CI**
NIM	0.0787	(0.0226, 0.1348)
SAU	0.0556	(0.0053, 0.1058)
**Visit 2**		
	NIM	SAU
NIM	41	0
SAU	2	15
No idea	112	145
**Bangs Index**	**Estimate**	**CI**
NIM	0.2516	(0.1788, 0.3245)
SAU	0.0938	(0.0486, 0.1389)
**Visit 3**		
	NIM	SAU
NIM	28	1
SAU	1	5
No idea	117	123
**Bangs Index**	**Estimate**	**CI**
NIM	0.1849	(0.1192, 0.2507)
SAU	0.031	(-0.0058, 0.0678)

Compliance with NIM and SAU

Supplementary Table 2 – compliance with NIM: a seven-category scale for compliance with the NIM intervention was developed and those families with compliance ratings of 5 or above were deemed "compliant" in a binary measure used for the CACE analysis. N.B. calculated for children not families.

Category	Detailed category	Numbers of children randomised to (GIFT)	Numbers of children randomised to (LIFT)
No compliance	1. Not offered GIFT/LIFT	35 (20.1%)	3 (6.1%)
	2. Consultation only (purpose to find out if worth pursuing assessment) (not relevant for LIFT)	14 (8.0%)	0 not relevant
	3. Offered GIFT but did not consent (not relevant for LIFT because court "consented" for them	19 (10.9%)	0 not relevant because all consented by judge
	4. Offered GIFT/LIFT but did not engage post consent (GIFT) or post court order (LIFT)	1 (0.6%)	3 (6.1%)
	Total non-compliers	69 (39.7%)	6 (12.2%)
Partial compliance	5. Offered and engaged in at least a partial assessment short of confident recommendation (GIFT or LIFT)	6 (3.4%)	1 (2.0%)
	6. Offered and engaged in full assessment but did not engage in treatment if it was offered (GIFT or LIFT) N.B. In LIFT, treatment might not have been offered to birth parents, even though a recommendation might have been made based on assessment. Treatment with other carers (e.g. foster or kinship may have occurred).	6 (3.4%)	29 (59.2%) Of these all had off-model treatment of foster or kinship carers post recommendation.
Full compliance	7. Offered and engaged in both assessment and trial of treatment (even if partial), including if offered assessment only because enough information for GIFT to make recommendation (GIFT or LIFT) For LIFT, it is still a 7 if the parent(s) were only offered assessment but this led to a confident decision to the court.	93 (53.4%) Of these, 26 had off-model treatment (i.e. beyond the recommendation date). 15 had off model treatment that lasted beyond the recommended 9 months.	13 (26.5%)
	Total compliers Glasgow and London	105 (60.3%)	43 (87.8%)
	Total cases randomised to GIFT or LIFT	174	49
	**Total compliance**	**148 (66.4%)**	
Total Cases		223 children from 180 families	

SAU: Of the 216 children whose families were randomised to Services as Usual, 156 started an assessment = 72.8%

The CACE analysis aims to assess if there is any effect of compliance on the primary SDQ outcome. The CACE model fitting was not possible for the full mixed effects regression as used for the primary SDQ analysis to enable direct comparisons. Instead, a simpler model without random effects and accounting for all data from all visits was fitted for the same ITT (ie FAS) population as used in primary analysis and the effect size was similar.

Placement Types

Supplementary Table 3 - Placement type at 2.5 years post-randomisation by intervention group

		Permanent Legal Order			No Permanence (temporary foster care or home with birth family with social work supervision)	Total
		Home	Adoption	Kinship/Special Guardianship Order		
Treatment condition and service recommendation	Services as usual					
	SAU recommendation- Home	11(39%)	1(4%)	1(4%)	15(54%)	28(100%)
	SAU recommendation - Away from Home	6(5%)	12(9%)	10(8%)	101(78%)	129(100%)
	SAU recommendation - no recommendation/Assessment not completed	9(28%)	4(13%)	1(3%)	18(56%)	32(100%)
		**55 (29%) SAU permanently placed**			134 (71%) SAU no permanence	
	NIM					
	NIM- Home	4(40%)	0(0%)	0(0%0	6(60%)	10(100%)
	NIM- Away from Home	2(2%)	15(12%)	10(8%)	96(78%)	123(100%)
	NIM- no recommendation	22(31%)	3(4%)	1(1%)	46(64%)	72(100%)
		**57 (28%) NIM permanently placed**			148 (72%) NIM no permanence	

Psychiatric Diagnoses at baseline

Supplementary Table 4

Psychiatric diagnoses based on child psychiatrist ratings of the Development and Wellbeing Assessment. Children were only eligible for a DAWBA is aged 2 or over. Although, in total, 40% of children had at least one psychiatric diagnosis, since many children had more than one diagnosis, numbers of diagnoses in this table add up to more than 40%.

	Baseline DAWBA Diagnoses present		
	Full sample (n=139)	Intervention group (n=75)	Services as usual group (n=64)
Anxiety Disorder	15	4	11
Depressive Disorder	3	3	0
Total emotional disorders		7	11
PTSD	5	2	3
Attachment Disorder	30	18	12
Total stress- and trauma-related disorders	35	20	15
Hyperkinetic Disorder	12	5	7
ASD/PDA	12	4	8
Total neurodevelopmental disorders	24	9	15
Total oppositional and conduct Disorders	16	10	6
Total Disorders (in 83 children)	93	46	47

Of the 139 children who had a baseline DAWBA, 83 children (60%) had no psychiatric diagnosis while 56 children (40%) had a least one psychiatric diagnosis.

Pre-specified subgroup analyses

Supplementary Table 5 - Subgroup and Interaction model for Site, with a model of pebtot~treat + visit + age + sex + fam.type + treat:visit + (1 | FirstSibFmt/subjid). All statistical tests were two-sided and not adjusted for multiple comparisons.

Model	NFamily	NChild	Effect	CI	Pval
SDQ Glasgow FAS, all present at randomisation (Number of families = 241, Number of children = 302‡)					
SDQ Glasgow FAS, all present at randomisation at interim visit	241	302	0.37	(-2.02, 2.77)	0.7605
SDQ Glasgow FAS, all present at randomisation at final visit	241	302	1.45	(-0.86, 3.77)	0.2173
SDQ London FAS, all present at randomisation (Number of families = 72, Number of children = 86‡)					
SDQ London FAS, all present at randomisation at interim visit	72	86	0.97	(-4.02, 6.10)	0.7014
SDQ London FAS, all present at randomisation at final visit	72	86	1.26	(-3.36, 6.01)	0.5926
Interaction					0.7347

Supplementary Table 6 - Subgroup and Interaction model for Sex, with a model of pebtot~treat + visit + age + fam.type + site + treat:visit + (1 | FirstSibFmt/subjid). All statistical tests were two-sided and not adjusted for multiple comparisons.

**Model**	**NFamily**	**NChild**	**Effect**	**CI**	**Pval**
SDQ Male FAS, all present at randomisation (Number of families = 187, Number of children = 211‡)					
SDQ Male FAS, all present at randomisation at interim visit	187	211	1.00	(-2.02, 4.05)	0.5163
SDQ Male FAS, all present at randomisation at final visit	187	211	0.12	(-2.82, 3.09)	0.9372
SDQ Female FAS, all present at randomisation (Number of families = 159, Number of children = 177‡)					
SDQ Female FAS, all present at randomisation at interim visit	159	177	-0.29	(-3.32, 2.76)	0.8522
SDQ Female FAS, all present at randomisation at final visit	159	177	2.96	(0.12, 5.82)	0.0419
Interaction					0.0217

BEST v2_1

Supplementary Table 7- Subgroup and Interaction model for Sex, with a model of pga~treat + visit + age + fam.type + site + treat:visit + (1 | subjid). All statistical tests were two-sided and not adjusted for multiple comparisons.

**Model**	**NFamily**	**NChild**	**Effect**	**CI**	**Pval**
PIRGAS Male FAS, all present at randomisation (Number of families = 117, Number of children = 128‡)					
PIRGAS Male FAS, all present at randomisation at interim visit	117	128	-0.08	(-7.57, 7.37)	0.9838
PIRGAS Male FAS, all present at randomisation at final visit	117	128	-6.61	(-14.19, 0.80)	0.0794
PIRGAS Female FAS, all present at randomisation (Number of families = 93, Number of children = 99‡)					
PIRGAS Female FAS, all present at randomisation at interim visit	93	99	-4.36	(-13.90, 5.27)	0.3677
PIRGAS Female FAS, all present at randomisation at final visit	93	99	3.61	(-5.10, 12.39)	0.4160
Interaction					0.1438

BEST v2_1

PIRGAS Sample Representativeness

Supplementary Table 8 – comparison of demographics in those children with and without a PIRGAS video

Variable	Statistic	Baseline PIRGAS completed			Baseline PIRGAS not completed		
		**All (N = 287)**	**NIM (N = 148)**	**CM (N = 139)**	**All (N = 177)**	**NIM (N = 88)**	**CM (N = 89)**
Age at start of study (years)†	N_{obs} (N_{miss})	287 (0)	148 (0)	139 (0)	177 (0)	88 (0)	89 (0)
	Mean (SD)	2.09 (1.65)	2.15 (1.65)	2.01 (1.65)	1.66 (1.64)	1.57 (1.61)	1.75 (1.68)
	Median (IQR)	1.93 [0.41, 3.41]	1.95 [0.54, 3.41]	1.91 [0.29, 3.37]	0.93 [0.16, 2.99]	0.77 [0.13, 2.83]	1.18 [0.20, 3.35]
	Range	(0.01, 5.46)	(0.01, 5.46)	(0.01, 5.40)	(0.02, 5.00)	(0.02, 5.00)	(0.04, 4.84)
Sex	N_{obs} (N_{miss})	287 (0)	148 (0)	139 (0)	172 (5)	86 (2)	86 (3)
Male	N (%)	150 (52.3%)	78 (52.7%)	72 (51.8%)	88 (51.2%)	53 (61.6%)	35 (40.7%)
Female	N (%)	137 (47.7%)	70 (47.3%)	67 (48.2%)	84 (48.8%)	33 (38.4%)	51 (59.3%)
SIMD (Decile)	N_{obs} (N_{miss})	272 (15)	140 (8)	132 (7)	137 (40)	71 (17)	66 (23)
1 - Most deprived	N (%)	171 (62.9%)	86 (61.4%)	85 (64.4%)	84 (61.3%)	49 (69.0%)	35 (53.0%)
Ethnicity‡	Nobs (Nmiss)	283 (4)	147 (1)	136 (3)	169 (3)	85 (1)	84 (2)
White	N (%)	235 (83.0%)	120 (81.6%)	115 (84.6%)	130 (76.9%)	61 (71.8%)	69 (82.1%)
Mixed	N (%)	24 (8.5%)	14 (9.5%)	10 (7.4%)	17 (10.1%)	9 (10.6%)	8 (9.5%)
Asian or Asian British	N (%)	12 (4.2%)	9 (6.1%)	3 (2.2%)	6 (3.6%)	3 (3.5%)	3 (3.6%)
Black or Black British	N (%)	12 (4.2%)	4 (2.7%)	8 (5.9%)	15 (8.9%)	11 (12.9%)	4 (4.8%)
Chinese or other ethnic group	N (%)	0 (0.0%)	0 (0.0%)	0 (0.0%)	1 (0.6%)	1 (1.2%)	0 (0.0%)
Care order‡	Nobs (Nmiss)	286 (1)	148 (0)	138 (1)	168 (4)	82 (4)	86 (0)
Voluntary	N (%)	123 (43.0%)	60 (40.5%)	63 (45.7%)	67 (39.9%)	33 (40.2%)	34 (39.5%)
Compulsary	N (%)	163 (57.0%)	88 (59.5%)	75 (54.3%)	101 (60.1%)	49 (59.8%)	52 (60.5%)
Number of previous placements‡	Nobs (Nmiss)	249 (38)	126 (22)	123 (16)	108 (64)	57 (29)	51 (35)
0	N (%)	213 (85.5%)	108 (85.7%)	105 (85.4%)	93 (86.1%)	51 (89.5%)	42 (82.4%)
1	N (%)	33 (13.3%)	16 (12.7%)	17 (13.8%)	14 (13.0%)	6 (10.5%)	8 (15.7%)
2	N (%)	3 (1.2%)	2 (1.6%)	1 (0.8%)	1 (0.9%)	0 (0.0%)	1 (2.0%)
SDQ-TD visit 1	Mean (SD)	12.6 (8.1)	11.4 (7.8)	14.1 (8.3)	13.1 (8.2)	12.6 (8.4)	13.5 (8.2)
SDQ-TD visit 2	Mean (SD)	11.8 (6.9)	11.8 (7.4)	11.9 (6.4)	12.7 (7.0)	11.8 (6.0)	13.5 (7.7)
SDQ -TD visit 3	Mean (SD)	11.2 (7.5)	11.8 (7.9)	10.6 (7.1)	11.4 (7.1)	10.9 (7.2)	12.0 (7.1)

Randomisation conditions

Supplementary Table 9a: Timelines of stages of Best Services Trial

STAGE	Feasibility RCT CSO funded	Bridging period NSPCC funded	NIHR funding/legal challenge to London randomisation	Definitive RCT with London site	End of recruitment	New end date with 2.5-year follow-up
TIME	Dec 2011-May 2015	June 2015-Dec 2015	Jan 2015-28.08.2017	29.08.2017	31.7.21	29.02.2024
RANDOMIS-ATION CONDITION	Consent > baseline > randomisation	Randomisation > consent > baseline		Consent > randomisation > baseline		
KEY CHALLENGES	Very high recruitment rate and delays to accessing services	Waiting lists due to teams treating non-RCT families		Potential for bias to be introduced through post-randomisation baseline assessment – necessary to comply with judicial requirements		

Supplementary Table 9b - Lower proportion converted from eligible to consented when participants know what service they have been randomised to.

Randomisation condition	Proportion consenting
Consent-randomisation	57.0%
Randomisation-consent	40.0%

Supplementary Table 9c - The proportion randomised to NIM is higher when participants are aware of the service they are randomised to prior to consenting

Randomisation condition	Proportion in SAU	Proportion in NIM	total
Consent-randomisation	165(51.0%)	160(49.2%)	325(100%)
Randomisation-consent	25(43.9%)	32(56.1%)	57(100%)

Study Measures

Supplementary Table 10a- Primary and Secondary Outcome Measures

	**Baseline**<br><br>***4-14 weeks post-care entry***	**Follow up 1**<br><br>***15 months post-care entry***	**Follow up 2**<br><br>***2.5 years post randomisation***	**Completeness at 2.5 years for those attending visit 3 (reported for Primary and secondary outcome measures)**	**Intraclass correlation coefficient**
SDQ<br><br>*To be completed for children aged 2 and over, according to age of child:*<br><br>*2-4y, 4-17y*<br>https://doi.org/10.1097/00004583-200111000-00015	✓	✓	✓	99.7%	0.38
PIR-GAS<br><br>*To be completed for all children*<br><br>https://doi.org/10.1046/j.1440-1819.2002.01044.x	✓	✓	✓	30.2%	0.17
PEDS-QL<br><br>*To be completed for all children, according to age of child:*<br><br>*1-12m, 13-24m, 2-4y, 5-7y*<br><br>https://doi.org/10.1586/14737167.5.6.705	✓	✓	✓	89.1%	0.35
TTPLS	Calculated through scrutiny of routine social care and legal system data			91.2%	

Supplementary Table 10b- Other Outcome Measures

	**Baseline**<br>***4-14 weeks post-care entry***	**Follow up 1**<br>***15 months post-care entry***	**Follow up 2**<br>***2.5 years post randomisation***
ITSEA<br>*To be completed for children aged 12-35 months*<br>https://doi.org/10.1002/imhj.20273	✓	✓	
DAI<br>*To be completed if child 12 months and over*	✓	✓	✓
DAWBA<br>*To be completed for children aged 2 and over, according to age of child: 2-4y, 4-17y*<br>https://doi.org/10.1111/j.1469-7610.2000.tb02345.x	✓	✓	✓
Service use questionnaire<br>*To be completed for all children*	✓	✓	✓
TIMB<br>*To be completed for all children*<br>https://doi.org/10.1177/10775595062912	✓	✓	✓
Observational Checklist for RAD<br>*To be completed if child 12 months and over*<br>https://doi.org/10.1177/25161032211050734	✓	✓(if not collected at baseline)	
Cognitive assessment<br>• *WPPSI III - 30-47 months,*<br>• *WPPSI III – 48 – 87 months*<br>*WISC IV – children + 87 months*	✓ (optional)	✓ (optional)	✓
RPQ<br>*To be completed if child 12 months and over*<br>https://doi.org/10.1177/1073191118797422	✓	✓	✓

Inter-rater reliability of PIRGAS and TIMB

Training for the two video-rated measures reported here, the Parent-infant-relationship-global-assessment-scale (PIRGAS) and the This is my baby assessment (TIMB) was conducted as follows? New raters initially had all of their PIRGAS and/or TIMB ratings double rated by and experienced raters. Discrepancies were brought to a conferencing meeting with at least one other experienced rater and these were discussed. After at least one further training iteration (double rating then conference), raters were allowed to be deemed experienced. Thereafter, a random 10 to 20% of each experienced rater’s ratings were randomly allocated by the Trial Manager to a second rater. These scores were sent to the Robertson Centre for assessment of inter-rater reliability (see Table below).

Supplementary Table 11

Correlation and completion rates of multiple scored TIMB and PIRGAS

Score	N_mult	N_final	Prop_mult	Correlation	CI	P_Val
PIRGAS	59	416	14.2	0.55	(0.34, 0.71)	<0.0001
TIMB Acceptance scale	98	933	10.5	0.61	(0.47, 0.72)	<0.0001
TIMB Commitment scale	98	933	10.5	0.51	(0.35, 0.64)	<0.0001
TIMB Awareness of Influence scale	98	933	10.5	0.71	(0.59, 0.79)	<0.0001

Any significant discrepancies (as defined in our standard operating procedures – see Crawford et al. Trials (2022) 23:122 https://doi.org/10.1186/s13063-022-06007-3) were brought to an approximately 3-monthly conference and an agreed score was submitted to the Robertson Centre based on that meeting. 26% of all PIRGAS videos were conferenced to resolve discrepancies and ensure a high-quality score was returned.

Statistical Analysis Plan

1. INTRODUCTION

1.1. STUDY BACKGROUND

Early intervention for maltreated infants can improve mental and physical health throughout life. Adverse experiences in early childhood increase the risk of mental health problems and physical disorders. The effect amplifies across the lifespan. Improving the mental health of young maltreated children is therefore beneficial to families as well as the population as a whole. An effective way to improve the mental health of young children is to improve existing relationships or to provide secure new ones, so that children can experience safe and nurturing care. The earlier this is achieved – ideally in the first year of life – the better the child recovers from maltreatment. Currently children often "revolve" between their maltreating birth family and temporary foster placements, which disrupts attachment and is detrimental to the child's wellbeing.

An intervention has been developed in New Orleans (Louisiana), aiming to improve the quality of permanent placement decisions. For each maltreated child under five all attachment relationships are assessed. Then a tailored intervention aiming to improve parent-child relationships is offered to each family. The success of this intervention feeds into the decision whether the child returns permanently to the birth family or whether it will be adopted. The implementation of this intervention led to an increased adoption rate and decreased maltreatment for those who stay with the birth family. Many mental health measures in these children are now very similar to those in the general population.

The trial began as a pilot trial in Glasgow. The data generated during the pilot phase will be integrated into the main trial.

1.2. STUDY OBJECTIVES

The research questions of the study are

- Is NIM effective in improving the mental health of maltreated infants and young children at 2.5 years follow-up compared to enhanced services as usual, Case Management (SAU)?
- Is NIM effective in improving the relationship between maltreated infants and young children and their primary caregiver, compared to enhanced services as usual, Case Management (SAU)?

- Does NIM effect more timely permanent placement decisions for maltreated children?
- Is NIM cost-effective in terms of both the mental health of the child and the longer-term impact on society as a whole?

1.3. Outcomes

1.3.1. Primary Outcome

The primary outcome of this study is child mental health measured by the Total Difficulties scale of the Strengths and Difficulties Questionnaire (SDQ).

1.3.2. Secondary Outcome

For the purposes of this SAP, the secondary outcomes of this study are:

1. Parent- or carer-child relationship, measured using the Parent-Infant Global Assessment Scale (PIR-GAS);
2. Child quality of life, measured using the Pediatric Quality of Life Inventory (Peds-QL);
3. Time to Permanent Placement.

1.3.3. Other Outcomes

Other study outcomes will be:

4. Other child mental health outcomes, measured using the Strengths and Difficulties Questionnaire (SDQ) sub-scales (Emotions, Conduct problems, Hyperactivity, Peer problems, and Prosocial);
5. Mental health diagnoses, measured using the Development and Wellbeing Assessment (DAWBA);
6. Attachment Disorder symptoms, measured using:
    a. the Disturbances of Attachment Interview (DAI) ("gold standard" for under 5s);
    b. the Relationship Problems Questionnaire ("gold standard" for children over age 5);
    c. the Observational Schedule for Reactive Attachment Disorder also known as the Waiting Room Observation (WRO);
7. Child emotional signalling, measured using the Emotional Signalling Scale (ESS);

8. Social-emotional development, measured using the Infant-Toddler Social-Emotional Assessment (ITSEA);
9. Child IQ, measured using the Wechsler Preschool and Primary Scale of Intelligence or Wechsler Intelligence Scale for Children (WPPSI / WISC IV);
10. Carers' long-term view of their relationship with the child, measured using the This Is My Baby (TIMB) questionnaire;
11. Service utilisation, measured using a Service Use Questionnaire (not covered in this SAP).
12. Repeat episodes of maltreatment (routine data linkage, not covered in this SAP).
13. Physical and mental health diagnoses (routine data linkage, not covered in this SAP).

1.4. Study Design

Two-arm, parallel-group, multicentre, randomised, controlled trial.

1.5. Randomisation and Consent

In the pilot phase between March 2012 to June 2015, families were randomised only after they had given consent and provided baseline data, which resulted in perceived delays in access to services. Between June 2015 until September 2017, the randomisation process was changed, so that eligible families were randomised as soon as the child(ren) entered care, prior to obtaining consent and baseline data. This did not improve delays in access to services, and in October 2017, the trial reverted to a system of randomisation after consent, though baseline data collection could take place after randomisation. This results in three randomisation systems:

1. Consent → Baseline → Randomisation
2. Randomisation → Consent → Baseline
3. Consent → Randomisation → Baseline

1.6. Sample Size and Power

Section 8.6 of the trial protocol states:

> *"The principal outcome will be the SDQ at 2.5 years follow-up for all children. A sample size of 462 will have 90% power to detect an effect size of 0.35, allowing for 25% loss to follow-up. Due to practical constraints, children from the same family cannot be randomised to different interventions, even if two children from the same family are referred at different times, therefore the study is effectively cluster randomised. The study will err on the side of caution and aim to recruit 462 families, though the analyses will be at the individual child level, with adjustment for clustering. Sensitivity analyses will look at analyses of one child per family, and*

still be well powered. This effect size equates to SDQ scores that are clinically significant."

1.7. Study Population

Families will be eligible for the trial if they have a child aged 0 to 60 months who enters care for reasons associated with maltreatment.

Detailed inclusion and exclusion criteria are given in the study protocol.

1.8. Statistical Analysis Plan (SAP)

1.8.1. SAP Objectives

The objective of this SAP is to describe the statistical analyses to be carried for the final analysis of the BEST trial. This SAP does not cover analysis of data obtained from linkage with routine data as this is likely to be available at a later time only.

1.8.2. General Principles

Baseline and follow-up demographic data will be summarised at all time points for all children in the trial.

Outcome data will be summarised at each time point at which it is measured and as changes over time where applicable.

Units of measurement will be indicated in the summary tables.

For those questionnaires that have different versions depending on the age of the child, results will be presented for each version separately, and if possible, presented using all versions on a common scale, e.g. by converting to percentiles of a normal population. Questionnaires that are completed for children who are not in the appropriate age range will be included in analyses in the first instance; sensitivity analyses may also be carried out with data collected using age-appropriate questionnaires only.

For outcomes measured at multiple time points, randomised groups will be compared using generalized linear mixed effects regression models, to account for clustering of outcomes within families, and for repeated measures of the outcome over time. The residual variance within each model will be assessed in blinded analyses and an appropriate model will be used for each outcome. Models will include fixed effects for randomised group, where possible the minimisation/stratification factors, time points (baseline, 15 months, 2.5 years, as appropriate), treatment-by-time interaction, child's age at time of data collection, and (where appropriate) version of questionnaire used, plus random effects for families and children, with a general covariance structure for the repeated measures. These models will be used to estimate the between-group differences at 15 months and 2.5 years from randomisation, with 95% confidence intervals and p-values. Should regression models encounter convergence difficulties when the actual randomisation codes are applied, then a simpler model will be applied;

all modelling decisions made after unblinding will be fully justified within the final statistical results.

For outcomes based on age-specific questionnaires, where it is possible to convert outcomes onto a common scale (such as percentiles of a normal population), similar models will be applied, without adjustment for the version of the questionnaire used.

Data from all children in each family who enter the study at the point of randomisation will be included in the final analyses. Sensitivity analyses will be carried out using one child from each family (the youngest child at the point of randomisation), and including all children who enter the study in each family (i.e. all who enter the study at the point of randomisation and also those who enter the study after randomisation). Missing outcome data will not be imputed.

The minimisation factors used are:

- age of the youngest child coming into care at the point of randomisation (<2, ≥2 years);
- number of children coming into care at the point of randomisation (1, or more than 1);
- birth family fluency in English;
- type of care (foster, kinship);
- study site.

Where possible, interaction models will be used to assess whether intervention effects vary between subgroups.

The level of significance for the primary outcome is 0.05. The statistical report will present p-values without adjustment for multiple comparisons.

1.8.3. Current Protocol

The study protocol at the time of writing this SAP is version 8.0, dated 14th December 2021.

1.8.4. Deviations To Those Specified In The Protocol

This SAP is consistent with the current protocol.

1.8.5. Software

The statistical software packages used will be R for Windows v4.0.1, SAS for Windows v9.2, or higher versions of these programs.

2. Analysis

2.1. Study Populations

The randomised population consists of all children who are considered eligible for the trial (at the time of randomisation) within families who are randomised.

The extended randomised population consists of all children who are considered to be eligible within families who are randomised, including children who come into care after randomisation.

The Full Analysis Set (FAS) consists of all children in the extended randomised population, for whom consent for participation in the trial is given, and for whom were not randomised in error, became ineligible, or subject to a legal withdrawal before data was collected.

If a family refuses the service they have been randomised to but still participates in the study they will be included in the FAS.

All analyses will be carried out on the FAS unless specified otherwise.

For each population, the number of families and the number of children will be given in total and by randomised group. Reasons for not being in the FAS will be summarised as available.

2.2. Baseline Characteristics

Summaries will be presented for the following baseline characteristics and measures:

- Demographics (at screening):
    - Age when entering care
    - Sex (Male / Female)
    - First language (English, Other)
    - Ethnicity (White, Mixed, Asian or Asian British, Black or Black British, Chinese or other ethnic group)
    - (S)IMD Quintile
    - Number of children randomised per family
    - Care order (Voluntary / Compulsory)
    - Number of previous placements
    - Reasons for entering care (multiple yes/no)
    - Entering care from home / hospital / Kinship care / Woman's aid / Other
    - Parental age
    - Abuse history (i.e. experience of physical, sexual, emotional abuse and neglect):
        - Adverse Childhood Experiences (ACES) questionnaire score (number of adverse childhood experiences)
        - Maltreatment Classification System (MCS) (individual maltreatment classifications and severity)

- Baseline values of outcome measures listed in Section 1.3.2

Baseline summaries of child-level data will be presented for all children, and for the youngest child at time of randomisation in each family. Baseline summaries will be presented overall and by randomised group.

Baseline summaries will also be provided by randomisation system in place at the time of randomisation.

2.3. Efficacy Outcomes

2.3.1. Primary Outcome

The primary outcome measure is the Total Difficulties scale of the SDQ at 2.5 years after entering care.

A single model over all three time points will be fitted, as described in Section 1.8.2. This model will not require adjustment for the version of questionnaire used. The primary intervention effect estimate will be taken as the interaction term between the intervention effect and the 2.5-year time point effect. This will be reported with a 95% confidence interval and p-value. The intervention effect estimate at the interim visit will be considered as a secondary analysis.

Blinded data analyses will be carried out during the trial, to determine whether data need to be transformed prior to analysis, or if alternative link or variance functions are required within the model.

CACE (Complier Average Causal Effect) analysis of the effect of compliance to intervention will be performed using a two-stage least squares regression approach for the primary outcome. Compliance will be defined according to a seven item scale, where scores of 4 or above will be considered as complying to study intervention.

2.3.2. Secondary Outcomes

Peds-QL and PIR-GAS will be analysed using the approach outlined in Section 1.8.2. Time to Permanent Placement (i.e. date of legal order, or date of return to birth family and removal of social work involvement) will be analysed using a Cox Proportional Hazards regression model, with adjustment for (where possible) minimisation factors.

2.4. Safety Outcomes

2.4.1. Serious Adverse Events

Serious adverse events (SAEs) will be summarised overall and by intervention group, for SAEs relating to study children, or their birth parents.

The seriousness criteria, severity, relationship to study procedures, expectedness, outcome and whether ongoing at the end of the study will be presented. The number of individuals experiencing at least one SAE will be summarised for all events. This will

be done for both children and birth parents. Similar summaries will be presented for number of families experiencing at least one SAE.

2.4.2. Withdrawal of Consent

The number and percentage of families who do not consent initially will be reported. The number and percentage of families who withdraw from the study, or parts of the study, will be reported. Reasons for withdrawal will be summarised or listed, as appropriate.

2.5. Subgroups

Where there is sufficient data, the following subgroups will be considered as part of exploratory analysis:

- study site;
- deprivation, as determined by (Scottish) Index of Multiple Deprivation quintiles;
- age of youngest eligible child coming into care at the point of randomisation (<2 / ≥2 years);
- number of eligible children coming into care at the point of randomisation (one / more than one child);
- birth family first language (English/Other);
- type of care (foster, kinship);
- sex;
- randomisation system in place at the time of randomisation.

The primary outcome will be summarised overall and by randomised group, separately within each subgroup, and the primary regression model will be extended to include intervention-by-subgroup interaction terms, to estimate intervention effects within subgroups, and to test for heterogeneity in intervention effects between subgroups.

2.6. Additional Analyses

Additional analyses will be agreed based on the results of the main SAP analysis and will be documented separately. These may include:

- sensitivity of main results to interruption due to the COVID pandemic.
- sensitivity of main results to alternative assumptions regarding missing outcome data;
- multiple imputation methods to impute data missing at random from questionnaires;
- analysis of routine data that needs to be linked to the study data, which will become available some months after the end of the trial;

3. Document History

This is v1.0 of the SAP for the final analysis of the BeST$^{?}$ trial, dated 2nd February 2024, and is the initial creation of this document.

4. Tables

Dummy tables will be produced during the development of the statistical analysis programs for review and feedback, and where possible will be reviewed prior to database lock.

5. Figures

Dummy figures will be produced during the development of the statistical analysis programs for review and feedback, and where possible will be reviewed prior to database lock.

6. Listings

Formal data listings of all SAEs and withdrawals will be included as part of the final statistical outputs. Derived datasets will be made available to the study team after the final statistical outputs have been produced.

Index children

Supplementary Table 11 – Treatment output of index/all child analysis, where the treatment effects (NIM minus SAU) at interim and final visits have been extracted from the full analysis output tables for SDQ, PEDSQL and PIRGAS analysis. PEDSQL uses logit transformed outcome.

Model	NFamily	NChild	Effect	CI	Pval
SDQ FAS, all present at randomisation at interim visit	313	388	0.45	(-1.72, 2.63)	0.6867
SDQ FAS, all present at randomisation at final visit	313	388	1.44	(-0.63, 3.53)	0.1728
SDQ all subjects at interim visit	315	406	0.47	(-1.67, 2.62)	0.6674
SDQ all subjects at final visit	315	406	1.41	(-0.64, 3.47)	0.1785
SDQ index children only at interim visit	310	310	-0.21	(-2.68, 2.29)	0.8688
SDQ index children only at final visit	310	310	0.48	(-1.86, 2.85)	0.6857
PedSQL FAS, all present at randomisation at interim visit	343	420	-0.02	(-0.23, 0.19)	0.8551
PedSQL FAS, all present at randomisation at final visit	343	420	-0.05	(-0.27, 0.16)	0.6299
PedSQL all subjects at interim visit	343	444	-0.04	(-0.25, 0.16)	0.6871
PedSQL all subjects at final visit	343	444	-0.08	(-0.29, 0.13)	0.4465
PedSQL index children only at interim visit	342	342	-0.05	(-0.28, 0.18)	0.6857
PedSQL index children only at final visit	342	342	0.00	(-0.23, 0.24)	0.9793
PIRGAS FAS, all present at randomisation at interim visit	194	227	-1.42	(-7.29, 4.47)	0.6354
PIRGAS FAS, all present at randomisation at final visit	194	227	-2.82	(-8.53, 2.84)	0.3284
PIRGAS all subjects at interim visit	195	238	-1.32	(-7.10, 4.47)	0.6528
PIRGAS all subjects at final visit	195	238	-2.62	(-8.22, 2.94)	0.3557
PIRGAS index children only at interim visit	188	188	-1.49	(-8.07, 5.11)	0.6563
PIRGAS index children only at final visit	188	188	-4.18	(-10.66, 2.28)	0.2049

Additional analyses

Sample size: We have now included the following detail in our Supplementary Materials, page 24: We examined study power in May 2021, at which stage there were 482 children randomised. Of those who had been in the study long enough, the retention rate at 2.5 years was 65.6%. Therefore, we projected 316 would provide primary outcome data at 2.5 years. At that point, the average cluster size was 1.239, and the ICC for the primary outcome at 2.5 years was 0.540, implying a design effect due to clustering of 1.13. The projected sample size of 158 per group would therefore be equivalent to 139 per group for an individually-randomised trial. This sample size would give 82.8% power to detect a standardised effect of 0.35.

By the end of the trial the SDQ ICC had reduced to 0.38. The retention rate improved towards the end of the study, with SDQ data available at 2.5 years for 366 children from 288 families, giving a design effect of 1.10. We therefore had more children than were projected, and a smaller design effect, both of which imply greater power than projected at the end of recruitment.

Supplementary Table 12 - FAS, Full output for coefficients of SDQ analysis

Comparison	Estimate	Standard Error	95% CI	P-value
NIM minus SAU *	-1.44	1.03	(-3.48, 0.60)	0.1647
Interim visit minus baseline*	-1.11	0.81	(-2.71, 0.48)	0.1723
Final visit minus baseline*	-2.41	0.86	(-4.11, -0.71)	0.0055
Age (years)	0.51	0.21	(0.11, 0.92)	0.0138
Multiple children families minus single-child families	1.94	0.73	(0.49, 3.38)	0.0087
Female minus Male	-1.14	0.62	(-2.36, 0.09)	0.0693
London minus Glasgow	-0.81	0.85	(-2.49, 0.87)	0.3449
NIM minus SAU at interim visit†	0.45	1.10	(-1.72, 2.63)	0.6867
NIM minus SAU at final visit†	1.44	1.06	(-0.63, 3.53)	0.1728

Supplementary Table 13- FAS, Full output for coefficients of logit-transformed PedSQL analysis

Comparison	Estimate	Standard Error	95% CI
NIM minus SAU *	0.01	0.09	(-0.17, 0.20)
Interim visit minus baseline*	0.28	0.09	(0.11, 0.44)
Final visit minus baseline*	0.38	0.10	(0.18, 0.58)
Age (years)	-0.12	0.04	(-0.20, -0.05)
Female minus Male	0.20	0.07	(0.06, 0.34)
Multiple children families minus single-child families	-0.12	0.08	(-0.27, 0.03)
London minus Glasgow	-0.02	0.09	(-0.19, 0.15)
Version for 12-24 months minus 0-12 months	-0.25	0.10	(-0.45, -0.05)
Version for 2-5 years minus 0-12 months	0.00	0.13	(-0.26, 0.26)
Version for 5-8 years minus 0-12 months	-0.10	0.22	(-0.53, 0.32)
NIM minus SAU at interim visit†	-0.02	0.11	(-0.23, 0.19)
NIM minus SAU at final visit†	-0.05	0.11	(-0.27, 0.16)

Supplementary Table 14- FAS, Full output for coefficients of PIRGAS analysis

Comparison	Estimate	Standard Error	95% CI
NIM minus SAU *	2.35	1.77	(-1.13, 5.84)
Interim visit minus baseline*	-0.79	2.12	(-4.96, 3.38)
Final visit minus baseline*	1.63	2.43	(-3.18, 6.46)
Age (years)	1.29	0.45	(0.40, 2.18)
Female minus Male	3.81	1.37	(1.11, 6.53)
Multiple children families minus single-child families	1.13	1.43	(-1.70, 3.94)
London minus Glasgow	-0.71	3.33	(-7.25, 5.88)
NIM minus SAU at interim visit†	-1.42	2.99	(-7.29, 4.47)
NIM minus SAU at final visit†	-2.82	2.88	(-8.53, 2.84)

Supplementary Table 15-FAS, Full output for Cox-ph models for time to permanent legal order

Variable	Test	Level	Events	HR (95% CI)	P-value
Time to permanent legal order, FAS	Treatment	SAU	55/189 (29.1%)	1.00 (-)	-
		NIM	57/205 (27.8%)	0.98 (0.68, 1.43)	0.9273
	Age at randomisation (years)	-	-	0.88 (0.78, 1.00)	0.0529
	Family type at randomisation	Solo	85/246 (34.6%)	1.00 (-)	-
		Multiple	27/148 (18.2%)	0.56 (0.36, 0.87)	0.0106
	Sex	Male	52/210 (24.8%)	1.00 (-)	-
		Female	60/184 (32.6%)	1.20 (0.83, 1.76)	0.3356
	Main site	Glasgow	60/316 (19.0%)	1.00 (-)	-
		London	52/78 (66.7%)	4.36 (2.96, 6.40)	<0.0001

Supplementary Table 16 (part 1)
Baseline details by SDQ completion at 2.5 years

Variable	Statistic	SDQ v3 completed			SDQ v3 not completed		
		**All (N = 351)**	**NIM (N = 184)**	**CM (N = 167)**	**All (N = 88)**	**NIM (N = 39)**	**CM (N = 49)**
Age at start of study (years)†	N_{obs} (N_{miss})	351 (0)	184 (0)	167 (0)	88 (0)	39 (0)	49 (0)
	Mean (SD)	2.05 (1.63)	2.06 (1.65)	2.04 (1.61)	1.74 (1.68)	1.69 (1.56)	1.78 (1.79)
	Median (IQR)	1.88 [0.42, 3.37]	1.84 [0.41, 3.36]	1.91 [0.44, 3.37]	1.11 [0.14, 3.41]	1.39 [0.12, 3.32]	1.11 [0.16, 3.74]
	Range	(0.01, 5.40)	(0.02, 5.38)	(0.01, 5.40)	(0.01, 5.19)	(0.01, 4.42)	(0.04, 5.19)
Sex	N_{obs} (N_{miss})	351 (0)	184 (0)	167 (0)	83 (5)	37 (2)	46 (3)
Male	N (%)	189 (53.8%)	105 (57.1%)	84 (50.3%)	41 (49.4%)	20 (54.1%)	21 (45.7%)
Female	N (%)	162 (46.2%)	79 (42.9%)	83 (49.7%)	42 (50.6%)	17 (45.9%)	25 (54.3%)
SIMD (Decile)	N_{obs} (N_{miss})	311 (40)	168 (16)	143 (24)	75 (13)	31 (8)	44 (5)
1 - Most deprived	N (%)	193 (62.1%)	103 (61.3%)	90 (62.9%)	44 (58.7%)	22 (71.0%)	22 (50.0%)
2	N (%)	55 (17.7%)	30 (17.9%)	25 (17.5%)	14 (18.7%)	6 (19.4%)	8 (18.2%)
3	N (%)	24 (7.7%)	16 (9.5%)	8 (5.6%)	6 (8.0%)	0 (0.0%)	6 (13.6%)
4	N (%)	14 (4.5%)	6 (3.6%)	8 (5.6%)	6 (8.0%)	2 (6.5%)	4 (9.1%)
5	N (%)	11 (3.5%)	7 (4.2%)	4 (2.8%)	3 (4.0%)	1 (3.2%)	2 (4.5%)
6	N (%)	4 (1.3%)	2 (1.2%)	2 (1.4%)	0 (0.0%)	0 (0.0%)	0 (0.0%)
7	N (%)	3 (1.0%)	1 (0.6%)	2 (1.4%)	1 (1.3%)	0 (0.0%)	1 (2.3%)
8	N (%)	1 (0.3%)	1 (0.6%)	0 (0.0%)	0 (0.0%)	0 (0.0%)	0 (0.0%)
9	N (%)	6 (1.9%)	2 (1.2%)	4 (2.8%)	0 (0.0%)	0 (0.0%)	0 (0.0%)
10 - Least Deprived	N (%)	0 (0.0%)	0 (0.0%)	0 (0.0%)	1 (1.3%)	0 (0.0%)	1 (2.3%)

†Age at start of study for a child is determined to be the age at randomisation for index children, and for non-index children will be whichever is later out of the time of randomisation and the time of enrolment
‡ Number of observations given out of the children with baseline case report form being available.

Supplementary Table 16 continued (part 2)
Baseline details by SDQ completion at 2.5 years

Variable	Statistic	SDQ v3 completed			SDQ v3 not completed		
		All (N = 351)	NIM (N = 184)	CM (N = 167)	All (N = 88)	NIM (N = 39)	CM (N = 49)
Randomisation schedule	N_{obs} (N_{miss})	351 (0)	184 (0)	167 (0)	88 (0)	39 (0)	49 (0)
Consent →Baseline →Randomisation	N (%)	129 (36.8%)	64 (34.8%)	65 (38.9%)	17 (19.3%)	5 (12.8%)	12 (24.5%)
Randomisation →Consent →Baseline	N (%)	40 (11.4%)	24 (13.0%)	16 (9.6%)	18 (20.5%)	12 (30.8%)	6 (12.2%)
Consent →Randomisation →Baseline	N (%)	182 (51.9%)	96 (52.2%)	86 (51.5%)	53 (60.2%)	22 (56.4%)	31 (63.3%)
Area	N_{obs} (N_{miss})	351 (0)	184 (0)	167 (0)	88 (0)	39 (0)	49 (0)
Glasgow	N (%)	271 (77.2%)	143 (77.7%)	128 (76.6%)	67 (76.1%)	31 (79.5%)	36 (73.5%)
London	N (%)	80 (22.8%)	41 (22.3%)	39 (23.4%)	21 (23.9%)	8 (20.5%)	13 (26.5%)
Site	N_{obs} (N_{miss})	351 (0)	184 (0)	167 (0)	88 (0)	39 (0)	49 (0)
Glasgow	N (%)	267 (76.1%)	141 (76.6%)	126 (75.4%)	66 (75.0%)	30 (76.9%)	36 (73.5%)
Croydon	N (%)	34 (9.7%)	19 (10.3%)	15 (9.0%)	13 (14.8%)	5 (12.8%)	8 (16.3%)
Tower Hamlets	N (%)	17 (4.8%)	8 (4.3%)	9 (5.4%)	4 (4.5%)	2 (5.1%)	2 (4.1%)
Renfrewshire	N (%)	4 (1.1%)	2 (1.1%)	2 (1.2%)	1 (1.1%)	1 (2.6%)	0 (0.0%)
Bromley	N (%)	17 (4.8%)	10 (5.4%)	7 (4.2%)	2 (2.3%)	0 (0.0%)	2 (4.1%)
Sutton	N (%)	4 (1.1%)	3 (1.6%)	1 (0.6%)	2 (2.3%)	1 (2.6%)	1 (2.0%)
Barking & Dagenham	N (%)	8 (2.3%)	1 (0.5%)	7 (4.2%)	0 (0.0%)	0 (0.0%)	0 (0.0%)
Newham	N (%)	0 (0.0%)	0 (0.0%)	0 (0.0%)	0 (0.0%)	0 (0.0%)	0 (0.0%)
Baseline child details CRF available	N_{obs} (N_{miss})	351 (0)	184 (0)	167 (0)	88 (0)	39 (0)	49 (0)
No	N (%)	0 (0.0%)	0 (0.0%)	0 (0.0%)	5 (5.7%)	2 (5.1%)	3 (6.1%)
Yes	N (%)	351 (100.0%)	184 (100.0%)	167 (100.0%)	83 (94.3%)	37 (94.9%)	46 (93.9%)

†Age at start of study for a child is determined to be the age at randomisation for index children, and for non-index children will be whichever is later out of the time of randomisation and the time of enrolment
‡ Number of observations given out of the children with baseline case report form being available.

Supplementary Table 16 continued (part 3)

Baseline details by SDQ completion at 2.5 years

Variable	Statistic	SDQ v3 completed			SDQ v3 not completed		
		All (N = 351)	NIM (N = 184)	CM (N = 167)	All (N = 88)	NIM (N = 39)	CM (N = 49)
Ethnicity‡	N_{obs} (N_{miss})	345 (6)	182 (2)	163 (4)	83 (0)	37 (0)	46 (0)
White	N (%)	275 (79.7%)	143 (78.6%)	132 (81.0%)	70 (84.3%)	29 (78.4%)	41 (89.1%)
Mixed	N (%)	34 (9.9%)	18 (9.9%)	16 (9.8%)	5 (6.0%)	3 (8.1%)	2 (4.3%)
Asian or Asian British	N (%)	13 (3.8%)	8 (4.4%)	5 (3.1%)	3 (3.6%)	2 (5.4%)	1 (2.2%)
Black or Black British	N (%)	22 (6.4%)	12 (6.6%)	10 (6.1%)	5 (6.0%)	3 (8.1%)	2 (4.3%)
Chinese or other ethnic group	N (%)	1 (0.3%)	1 (0.5%)	0 (0.0%)	0 (0.0%)	0 (0.0%)	0 (0.0%)
Care order‡	N_{obs} (N_{miss})	347 (4)	180 (4)	167 (0)	82 (1)	37 (0)	45 (1)
Voluntary	N (%)	142 (40.9%)	74 (41.1%)	68 (40.7%)	38 (46.3%)	14 (37.8%)	24 (53.3%)
Compulsary	N (%)	205 (59.1%)	106 (58.9%)	99 (59.3%)	44 (53.7%)	23 (62.2%)	21 (46.7%)
Number of previous placements‡	N_{obs} (N_{miss})	271 (80)	144 (40)	127 (40)	70 (13)	31 (6)	39 (7)
0	N (%)	231 (85.2%)	124 (86.1%)	107 (84.3%)	59 (84.3%)	27 (87.1%)	32 (82.1%)
1	N (%)	37 (13.7%)	18 (12.5%)	19 (15.0%)	10 (14.3%)	4 (12.9%)	6 (15.4%)
2	N (%)	3 (1.1%)	2 (1.4%)	1 (0.8%)	1 (1.4%)	0 (0.0%)	1 (2.6%)
Entered care from‡	N_{obs} (N_{miss})	341 (10)	178 (6)	163 (4)	83 (0)	37 (0)	46 (0)
Hospital	N (%)	73 (21.4%)	36 (20.2%)	37 (22.7%)	30 (36.1%)	12 (32.4%)	18 (39.1%)
Home	N (%)	239 (70.1%)	128 (71.9%)	111 (68.1%)	45 (54.2%)	19 (51.4%)	26 (56.5%)
Kinship care	N (%)	16 (4.7%)	10 (5.6%)	6 (3.7%)	7 (8.4%)	5 (13.5%)	2 (4.3%)
Womans Aid	N (%)	1 (0.3%)	0 (0.0%)	1 (0.6%)	0 (0.0%)	0 (0.0%)	0 (0.0%)
Other	N (%)	12 (3.5%)	4 (2.2%)	8 (4.9%)	1 (1.2%)	1 (2.7%)	0 (0.0%)

†Age at start of study for a child is determined to be the age at randomisation for index children, and for non-index children will be whichever is later out of the time of randomisation and the time of enrolment

‡ Number of observations given out of the children with baseline case report form being available.

Supplementary Table 16 continued (part 4)
Baseline details by SDQ completion at 2.5 years

SDQ v3 completed			SDQ v3 not completed				
**All (N = 351)**	**NIM (N = 184)**	**CM (N = 167)**	**All (N = 88)**		**NIM (N = 39)**	**CM (N = 49)**	
Number of reasons for entering care	N_{obs} (N_{miss})	351 (0)	184 (0)	167 (0)	83 (0)	37 (0)	46 (0)
0	N (%)	28 (8.0%)	14 (7.6%)	14 (8.4%)	5 (6.0%)	3 (8.1%)	2 (4.3%)
1	N (%)	77 (21.9%)	38 (20.7%)	39 (23.4%)	18 (21.7%)	7 (18.9%)	11 (23.9%)
2	N (%)	108 (30.8%)	57 (31.0%)	51 (30.5%)	26 (31.3%)	12 (32.4%)	14 (30.4%)
3	N (%)	96 (27.4%)	53 (28.8%)	43 (25.7%)	21 (25.3%)	9 (24.3%)	12 (26.1%)
≥4	N (%)	42 (12.0%)	22 (12.0%)	20 (12.0%)	13 (15.7%)	6 (16.2%)	7 (15.2%)
Reasons for entering care (multiple options may apply)							
Neglect	N (%)	191 (54.4%)	96 (52.2%)	95 (56.9%)	37 (44.6%)	18 (48.6%)	19 (41.3%)
Domestic abuse	N (%)	93 (26.5%)	48 (26.1%)	45 (26.9%)	31 (37.3%)	12 (32.4%)	19 (41.3%)
Physical abuse	N (%)	50 (14.2%)	29 (15.8%)	21 (12.6%)	12 (14.5%)	5 (13.5%)	7 (15.2%)
Substance abuse	N (%)	107 (30.5%)	53 (28.8%)	54 (32.3%)	24 (28.9%)	14 (37.8%)	10 (21.7%)
Parental mental health issue	N (%)	97 (27.6%)	60 (32.6%)	37 (22.2%)	25 (30.1%)	13 (35.1%)	12 (26.1%)
Alcohol abuse	N (%)	68 (19.4%)	36 (19.6%)	32 (19.2%)	13 (15.7%)	7 (18.9%)	6 (13.0%)
Homeless	N (%)	9 (2.6%)	7 (3.8%)	2 (1.2%)	3 (3.6%)	1 (2.7%)	2 (4.3%)
Inadequate home conditions	N (%)	42 (12.0%)	22 (12.0%)	20 (12.0%)	9 (10.8%)	5 (13.5%)	4 (8.7%)
Emotional abuse	N (%)	42 (12.0%)	30 (16.3%)	12 (7.2%)	13 (15.7%)	9 (24.3%)	4 (8.7%)
Parental chaotic lifestyle	N (%)	34 (9.7%)	16 (8.7%)	18 (10.8%)	10 (12.0%)	6 (16.2%)	4 (8.7%)
Inadequate attendance for antenatal care	N (%)	11 (3.1%)	4 (2.2%)	7 (4.2%)	6 (7.2%)	2 (5.4%)	4 (8.7%)
Non-engagement with social work	N (%)	48 (13.7%)	23 (12.5%)	25 (15.0%)	17 (20.5%)	5 (13.5%)	12 (26.1%)
Other	N (%)	8 (2.3%)	3 (1.6%)	5 (3.0%)	4 (4.8%)	1 (2.7%)	3 (6.5%)

Reporting checklist for randomised trial

Based on the CONSORT guidelines.

Instructions to authors

Complete this checklist by entering the page numbers from your manuscript where readers will find each of the items listed below.

Schulz KF, Altman DG, Moher D, for the CONSORT Group. CONSORT 2010 Statement: updated guidelines for reporting parallel group randomised trials

		Reporting Item	Page Number N.B. main text unless specified as "Methods"
**Title and Abstract**			
Title	#1a	Identification as a randomized trial in the title.	1
Abstract	#1b	Structured summary of trial design, methods, results, and conclusions	2
**Introduction**			
Background and objectives	#2a	Scientific background and explanation of rationale	4
Background and objectives	#2b	Specific objectives or hypothesis	7
**Methods**			
Trial design	#3a	Description of trial design (such as parallel, factorial) including allocation ratio.	24
Trial design	#3b	Important changes to methods after trial commencement (such as eligibility criteria), with reasons	25
Participants	#4a	Eligibility criteria for participants	Methods 1
Participants	#4b	Settings and locations where the data were collected	Methods 1
Interventions	#5	The experimental and control interventions for each group with sufficient details to	4-7

		allow replication, including how and when they were actually administered	
Outcomes	#6a	Completely defined prespecified primary and secondary outcome measures, including how and when they were assessed	26
Outcomes	#6b	Any changes to trial outcomes after the trial commenced, with reasons	N/A
Sample size	#7a	How sample size was determined.	29
Sample size	#7b	When applicable, explanation of any interim analyses and stopping guidelines	N/A
Randomization - Sequence generation	#8a	Method used to generate the random allocation sequence.	10, 25
Randomization - Sequence generation	#8b	Type of randomization; details of any restriction (such as blocking and block size)	25
Randomization - Allocation concealment mechanism	#9	Mechanism used to implement the random allocation sequence (such as sequentially numbered containers), describing any steps taken to conceal the sequence until interventions were assigned	25
Randomization - Implementation	#10	Who generated the allocation sequence, who enrolled participants, and who assigned participants to interventions	25
Blinding	#11a	If done, who was blinded after assignment to interventions (for example, participants, care providers, those assessing outcomes) and how.	25
Blinding	#11b	If relevant, description of the similarity of interventions	N/A
Statistical methods	#12a	Statistical methods used to compare groups for primary and secondary outcomes	29

Statistical methods	#12b	Methods for additional analyses, such as subgroup analyses and adjusted analyses	29 and supplementary materials page 14
**Results**			
Participant flow diagram (strongly recommended)	#13a	For each group, the numbers of participants who were randomly assigned, received intended treatment, and were analysed for the primary outcome	28
Participant flow	#13b	For each group, losses and exclusions after randomization, together with reason	28
Recruitment	#14a	Dates defining the periods of recruitment and follow-up	10
Recruitment	#14b	Why the trial ended or was stopped	10
Baseline data	#15	A table showing baseline demographic and clinical characteristics for each group	10, 22
Numbers analysed	#16	For each group, number of participants (denominator) included in each analysis and whether the analysis was by original assigned groups	10
Outcomes and estimation	#17a	For each primary and secondary outcome, results for each group, and the estimated effect size and its precision (such as 95% confidence interval)	11,23
Outcomes and estimation	#17b	For binary outcomes, presentation of both absolute and relative effect sizes is recommended	N/A
Ancillary analyses	#18	Results of any other analyses performed, including subgroup analyses and adjusted analyses, distinguishing pre-specified from exploratory	12,13
Harms	#19	All important harms or unintended effects in each group (For specific guidance see CONSORT for harms)	11, 12

**Discussion**			
Limitations	#20	Trial limitations, addressing sources of potential bias, imprecision, and, if relevant, multiplicity of analyses	16
Generalisability	#21	Generalisability (external validity, applicability) of the trial findings	13,14
Interpretation	#22	Interpretation consistent with results, balancing benefits and harms, and considering other relevant evidence	13-15
Registration	#23	Registration number and name of trial registry	2
**Other information**			
Interpretation	#22	Interpretation consistent with results, balancing benefits and harms, and considering other relevant evidence	23
Registration	#23	Registration number and name of trial registry	2
Protocol	#24	Where the full trial protocol can be accessed, if available	24 and Supplementary Materials page 18
Funding	#25	Sources of funding and other support (such as supply of drugs), role of funders	17,18

None The CONSORT checklist is distributed under the terms of the Creative Commons Attribution License CC-BY. This checklist can be completed online using https://www.goodreports.org/, a tool made by the EQUATOR Network in collaboration with Penelope.ai
