# Supplementary material for: Infant mental health services for birth and foster families of maltreated pre-school children in foster care (BeST?): a cluster-randomized phase 3 clinical effectiveness trial
Source: Nat Med. 2025 May 1;31(5):1617–25. doi: 10.1038/s41591-025-03534-9 (PMC12092239; doi:10.1038/s41591-025-03534-9)
Supplement: Supplementary file 2 — Reporting Summary [file 41591_2025_3534_MOESM2_ESM.pdf]

nature portfolio

Corresponding author(s): Helen Minnis

Last updated by author(s): 18.12.24

Reporting Summary

Nature Portfolio wishes to improve the reproducibility of the work that we publish. This form provides structure for consistency and transparency in reporting. For further information on Nature Portfolio policies, see our Editorial Policies and the Editorial Policy Checklist.

Statistics

For all statistical analyses, confirm that the following items are present in the figure legend, table legend, main text, or Methods section.

n/a	Confirmed	
☐	☒	The exact sample size (*n*) for each experimental group/condition, given as a discrete number and unit of measurement
☐	☒	A statement on whether measurements were taken from distinct samples or whether the same sample was measured repeatedly
☐	☒	The statistical test(s) used AND whether they are one- or two-sided<br>*Only common tests should be described solely by name; describe more complex techniques in the Methods section.*
☐	☒	A description of all covariates tested
☐	☒	A description of any assumptions or corrections, such as tests of normality and adjustment for multiple comparisons
☐	☒	A full description of the statistical parameters including central tendency (e.g. means) or other basic estimates (e.g. regression coefficient) AND variation (e.g. standard deviation) or associated estimates of uncertainty (e.g. confidence intervals)
☐	☒	For null hypothesis testing, the test statistic (e.g. *F*, *t*, *r*) with confidence intervals, effect sizes, degrees of freedom and *P* value noted<br>*Give P values as exact values whenever suitable.*
☒	☐	For Bayesian analysis, information on the choice of priors and Markov chain Monte Carlo settings
☒	☐	For hierarchical and complex designs, identification of the appropriate level for tests and full reporting of outcomes
☐	☒	Estimates of effect sizes (e.g. Cohen's *d*, Pearson's *r*), indicating how they were calculated

Our web collection on statistics for biologists contains articles on many of the points above.

Software and code

Policy information about availability of computer code

Data collection	No software was used for data collection.
Data analysis	The primary analysis was conducted in R version 4.1.1 (2021-08-10) for Windows (with lme4 and survival for TTPLS)

For manuscripts utilizing custom algorithms or software that are central to the research but not yet described in published literature, software must be made available to editors and reviewers. We strongly encourage code deposition in a community repository (e.g. GitHub). See the Nature Portfolio guidelines for submitting code & software for further information.

Data

Policy information about availability of data

All manuscripts must include a data availability statement. This statement should provide the following information, where applicable:

- Accession codes, unique identifiers, or web links for publicly available datasets
- A description of any restrictions on data availability
- For clinical datasets or third party data, please ensure that the statement adheres to our policy

Although we aim for an open science approach to requests for study data, some controls regarding access is necessary because of the high sensitivity of this cohort in which the great majority of participants have experienced severe early adversities including abuse and neglect. Because of the modest numbers in some subgroups, care will need to be taken not to inadvertently identify individuals. Data requests will be considered by the Trial Management Group (TMG), which includes representatives of the sponsor, the University of Glasgow, senior investigators independent of the research team and the chief investigator. The TMG will

take account of the scientific rationale, ethics, logistics and resource implications. Data access requests should be initially submitted by email to the chief investigator (HM, corresponding author). It should usually be possible to convene the Trial Management Group within three months and we will endeavour to make decisions regarding data access within this timeframe. The source data include the de-identified numerical data used for the statistical analyses. Data access will be provided through the secure analytical platform of the Robertson Centre for Biostatistics. This secure platform enables access to de-identified data for analytical purposes, without the possibility of removing the data from the server. Requests for transfer of de-identified data will be considered by the Steering Group, and, if approved, a collaboration agreement would be expected. The TMG will consider any cost implications, and cost recovery would be expected on a not-for-profit basis.

Research involving human participants, their data, or biological material

Policy information about studies with human participants or human data. See also policy information about sex, gender (identity/presentation), and sexual orientation and race, ethnicity and racism.

Reporting on sex and gender	We have used the term "sex" throughout
Reporting on race, ethnicity, or other socially relevant groupings	We used ethnicity terminology as in the UK census. Participants were invited to classify themselves during the baseline research assessment. Classifiers were as follows:<br>Ethnicity<br>White<br>Mixed<br>Asian or Asian British<br>Black or Black British<br>Other ethnic group
Population characteristics	The participants are infants and pre-school children aged 0-5 who have experienced maltreatment and who have been placed in foster care - and their birth and foster families.
Recruitment	We used a novel recruitment and retention method: experienced social workers (ex-team-leaders) screened social care records for eligibility and conducted information and consent meetings with interested participants. We approached every eligible family (our social worker recruiters had access to data on the entire target population) so did all we could to avoid selection bias.
Ethics oversight	The trial was approved by West of Scotland Research Ethics Service, Committee 3 (15/WS/0280)

Note that full information on the approval of the study protocol must also be provided in the manuscript.

Field-specific reporting

Please select the one below that is the best fit for your research. If you are not sure, read the appropriate sections before making your selection.

☐ Life sciences ☒ Behavioural & social sciences ☐ Ecological, evolutionary & environmental sciences

For a reference copy of the document with all sections, see nature.com/documents/nr-reporting-summary-flat.pdf

Behavioural & social sciences study design

All studies must disclose on these points even when the disclosure is negative.

Study description	Quantitative multi-site, pragmatic, parallel group, single-blind (researchers blind to arm of trial), cluster randomised controlled superiority trial with an allocation ratio of 1:1 to compare the New Orleans Intervention Model for infant mental health, (NIM) with social work services-as-usual.
Research sample	The trial was conducted in two large UK cities, both containing areas of material deprivation. The study settings were social services in the local authorities/Boroughs feeding into the two trial sites: Greater Glasgow and Clyde (Scotland) comprising Glasgow City Council and Renfrewshire Council; and London (England), including the Boroughs of Croydon, Tower Hamlets, Sutton, Bromley and Barking & Dagenham. The participants are infants and pre-school children aged 0-5 who had experienced maltreatment and who have been placed in foster care - and their birth and foster families in London and Glasgow. For the Glasgow cohort (n= 338 children, broadly representative of the general population of looked after children in Glasgow
Sampling strategy	We took a total sample approach: all eligible families were approached in the study sites by experienced social workers (ex-team-leaders) who screened social care records for eligibility and conducted information and consent meetings with interested participants. The sample size was calculated by the Glasgow Clinical Trials Unit (CTU). Initially, the target sample size was 462 to achieve 90% power to detect and effect size of 0.35 SD on SDQ-TD with a loss to follow-up at 2.5 years of 25% 28. With no information as to the likely degree of clustering of the primary outcome within families, we aimed to recruit 462 families, even though the unit of analysis would be the individual child. Although we later decided to constrain the sample size for financial reasons 28, follow-up rates proved better than expected and the trial was well powered. Given the SD of SDQ-TD observed in the trial at 2.5 years, a 0.35 SD difference equates to a clinically meaningful 2.5 point difference in the 20-item SDQ-TD scale.
Data collection	Each measure was collected following standard operating proceedures included in training documentation and the study manual. All research nurses or psychologists collecting data were trained in administering study measures and practiced administration of

measures with each other prior to conducting research assessments. All data was collected using standardised paper case report forms (CRF) based on the individual instruments being used. These were version controlled. Only the data collector and participant(s) were present during data collection. Masking was assured for two secondary outcome measures, Time to Permanent Legal Status (TTPLS) and Parent-infant relationship global assessment scale (PIRGAS) because data were collected (TTPLS) and rated (PIRGAS) by individuals with no contact with participants or other trial procedures. We used Bang's Unblinding Index to examine our success with masking.

Timing	Data were collected between January 2012 and July 2021 and retention (final follow-up visits) were completed by December 2023.
Data exclusions	25 families were excluded after randomisation, according to our pre-specified exclusion criteria, because the parenting capacity assessment was no longer required, usually because a Judge (England) or Sheriff (Scotland) adjudicated that maltreatment had not occurred.
Non-participation	93 families declined participation.
Randomization	Random allocation was performed, through a web portal requiring a login and password with access rights allocated to relevant Clinical Trials Unit staff, using a mixed minimisation/randomisation method, stratified within study site using an a priori schedule for each site in blocks of 10. Minimisation factors were those likely to impact delivery of NIM: study site, age of youngest child coming into care at randomisation (<2/≥2 years), number of children coming into care at randomisation (1/>1), birth family fluency in English, and type of care (i.e. foster or kinship, i.e. with extended family).

Reporting for specific materials, systems and methods

We require information from authors about some types of materials, experimental systems and methods used in many studies. Here, indicate whether each material, system or method listed is relevant to your study. If you are not sure if a list item applies to your research, read the appropriate section before selecting a response.

Materials & experimental systems

n/a	Involved in the study
☒	☐ Antibodies
☒	☐ Eukaryotic cell lines
☒	☐ Palaeontology and archaeology
☒	☐ Animals and other organisms
☐	☒ Clinical data
☒	☐ Dual use research of concern
☒	☐ Plants

Methods

n/a	Involved in the study
☒	☐ ChIP-seq
☒	☐ Flow cytometry
☒	☐ MRI-based neuroimaging

Clinical data

Policy information about clinical studies
All manuscripts should comply with the ICMJE guidelines for publication of clinical research and a completed CONSORT checklist must be included with all submissions.

Clinical trial registration	ClinicalTrials.gov registration: NCT02653716
Study protocol	https://doi.org/10.1186/s13063-022-06007-3
Data collection	The trial was conducted in two large UK cities, both containing areas of material deprivation. The study settings were social services in the local authorities/Boroughs feeding into the two trial sites: Greater Glasgow and Clyde (Scotland) comprising Glasgow City Council and Renfrewshire Council; and London (England), including the Boroughs of Croydon, Tower Hamlets, Sutton, Bromley and Barking & Dagenham. The participants are infants and pre-school children aged 0-5 who had experienced maltreatment and who have been placed in foster care - and their birth and foster families in London and Glasgow. For the Glasgow cohort (n= 338 children, broadly representative of the general population of looked after children in Glasgow Each measure was collected following standard operating proceeures included in training documentation and the study manual. All research nurses or psychologists collecting data were trained in administering study measures and practiced administration of measures with each other prior to conducting research assessments. All data was collected using standardised paper case report forms (CRF) based on the individual instruments being used. These were version controlled. Only the data collector and participant(s) were present during data collection. Masking was assured for two secondary outcome measures, Time to Permanent Legal Status (TTPLS) and Parent-infant relationship global assessment scale (PIRGAS) because data were collected (TTPLS) and rated (PIRGAS) by individuals with no contact with participants or other trial procedures. We used Bang's Unblinding Index to examine our success with masking. Data were collected between January 2012 and July 2021 and retention (final follow-up visits) were completed by December 2023.
Outcomes	Because NIM aims to improve the mental health of the infant or pre-school child by offering relationship-focused interventions making a recommendation to the legal system about the child's permanent placement (Figure 1), primary and secondary outcome measures focused on mental health/quality of life, relationship functioning and permanency. The primary outcome measure was the 20-item Strengths and Difficulties Questionnaire Total Difficulties Scale (SDQ-TD) at 2.5 years after study entry, assessed at each site. The SDQ is a mental health screening questionnaire for 2-16-year-olds, completed by the primary caregiver, with 25 items in 5

subscales; emotional symptoms; conduct problems; hyperactivity/inattention; peer relationship problems and prosocial behaviour. The SDQ-TD does not include the prosocial subscale and is 20-items. Higher SDQ-TD scores indicate poorer child behaviour 29.
Secondary outcome measures were:
o Child quality of life (Pediatric Quality of Life Inventory, PedsQL) 30,
o Time taken to permanent legal status (TTPLS) being achieved authorising the child to be placed in permanent care (return home without social work oversight or adoption) 28, and
o Quality of the child's relationship with the primary caregiver (Parent-infant Global Assessment Scale, PIRGAS). For PIRGAS, a short playtime and mealtime video was conducted in the research clinic and independently rated by specifically trained raters 31
Study measures are detailed and referenced in Supplementary Table 10. Because NIM aims to improve the mental health of the infant or pre-school child by offering relationship-focused interventions making a recommendation to the legal system about the child's permanent placement (Figure 1), primary and secondary outcome measures focused on mental health/quality of life, relationship functioning and permanency. The primary outcome measure was the 20-item Strengths and Difficulties Questionnaire Total Difficulties Scale (SDQ-TD) at 2.5 years after study entry, assessed at each site. The SDQ is a mental health screening questionnaire for 2-16-year-olds, completed by the primary caregiver, with 25 items in 5 subscales; emotional symptoms; conduct problems; hyperactivity/inattention; peer relationship problems and prosocial behaviour. The SDQ-TD does not include the prosocial subscale and is 20-items. Higher SDQ-TD scores indicate poorer child behaviour 29.
Secondary outcome measures were:
o Child quality of life (Pediatric Quality of Life Inventory, PedsQL) 30,
o Time taken to permanent legal status (TTPLS) being achieved authorising the child to be placed in permanent care (return home without social work oversight or adoption) 28, and
o Quality of the child's relationship with the primary caregiver (Parent-infant Global Assessment Scale, PIRGAS). For PIRGAS, a short playtime and mealtime video was conducted in the research clinic and independently rated by specifically trained raters 31
Study measures are detailed and referenced in Supplementary Table 10. Because NIM aims to improve the mental health of the infant or pre-school child by offering relationship-focused interventions making a recommendation to the legal system about the child's permanent placement (Figure 1), primary and secondary outcome measures focused on mental health/quality of life, relationship functioning and permanency. The primary outcome measure was the 20-item Strengths and Difficulties Questionnaire Total Difficulties Scale (SDQ-TD) at 2.5 years after study entry, assessed at each site. The SDQ is a mental health screening questionnaire for 2-16-year-olds, completed by the primary caregiver, with 25 items in 5 subscales; emotional symptoms; conduct problems; hyperactivity/inattention; peer relationship problems and prosocial behaviour. The SDQ-TD does not include the prosocial subscale and is 20-items. Higher SDQ-TD scores indicate poorer child behaviour.
Secondary outcome measures were: Child quality of life (Pediatric Quality of Life Inventory, PedsQL); Time taken to permanent legal status (TTPLS) being achieved authorising the child to be placed in permanent care (return home without social work oversight or adoption) and; quality of the child's relationship with the primary caregiver (Parent-infant Global Assessment Scale, PIRGAS). For PIRGAS, a short playtime and mealtime video was conducted in the research clinic and independently rated by specifically trained raters.
Secondary outcome measures were:
o Child quality of life (Pediatric Quality of Life Inventory, PedsQL) 30,
o Time taken to permanent legal status (TTPLS) being achieved authorising the child to be placed in permanent care (return home without social work oversight or adoption) 28, and
o Quality of the child's relationship with the primary caregiver (Parent-infant Global Assessment Scale, PIRGAS). For PIRGAS, a short playtime and mealtime video was conducted in the research clinic and independently rated by specifically trained raters 31

Plants

Seed stocks

N/A

Novel plant genotypes

N/A

Authentication

N/A
